# Enhanced Piezoelectricity in Sustainable-By-Design Chitosan Nanocomposite Soft Thin Films for Green Sensors

**DOI:** 10.1021/acsnano.4c12855

**Published:** 2025-08-18

**Authors:** Jacopo Nicoletti, Leonardo Puppulin, Julie Routurier, Saimir Frroku, Nouha Loudhaief, Claudia Crestini, Alvise Perosa, Maurizio Selva, Matteo Gigli, Michele Back, Pietro Riello, Domenico De Fazio, Giovanni Antonio Salvatore

**Affiliations:** † 19047 Ca’ Foscari University of Venice, Department of Molecular Science and Nanosystems, Via Torino 155, Venezia 30172, Italy; ‡ Université de Haute-Alsace (UHA), Ecole de Chimie de Mulhouse (ENSCMu), 3 Rue Alfred Werner, Mulhouse 68200, France

**Keywords:** piezoelectricity, chitosan, nanocrystals, biowaste-upcycling, biopolymers, piezoresponse force microscopy (PFM)

## Abstract

Piezoelectricity, the generation of an electric charge in response to mechanical stress, is a key property in both natural and synthetic materials. This study significantly boosts the piezoelectric response of chitosan, a biodegradable biopolymer, by integrating chitin/surface-deacetylated chitin nanocrystals into natural chitosan-based thin films. The resulting materials, produced in our laboratories, achieve *d*_33_ values of up to 18.7 ± 1.1 pm V^–1^, a marked improvement over 8.9 ± 0.6 pm V^–1^ observed in pure chitosan films, thanks to the increased crystallinity provided by the nanocrystals. We utilize piezoresponse force microscopy (PFM) to accurately measure the *d*_33_ coefficient, employing an engineered extraction method that eliminates the electrostatic contribution, which can overestimate the piezoelectric response. This work introduces a soft, elastomer-like piezoelectric thin film entirely derived from upcycled biowaste. The chitosan-based films combine high elasticity (up to 40% strain) with a low Young’s modulus (∼100 MPa), closely mimicking soft biological tissues. Unlike previously reported piezoelectric materials, which typically rely on synthetic or inorganic components, our films are fully biobased and mechanically compliant, making them ideal for applications in prosthetics, wearable devices, soft robotics, and sustainable energy harvesting.

## Graphical abstract

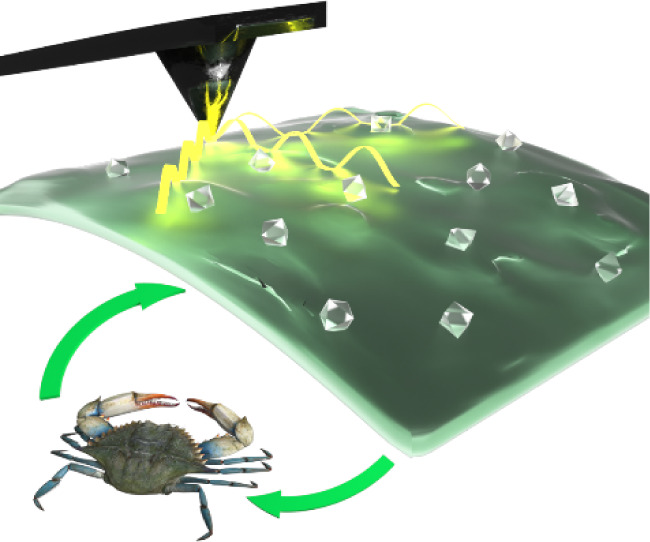

## Introduction

Piezoelectricity is the ability of certain materials to generate an electric charge in response to mechanical stress. It arises from an asymmetric crystal structure that polarizes the material when subjected to mechanical deformation. Piezoelectricity is a phenomenon widely observed in nature, particularly in plants and animals.^1–5^ In plants, piezoelectric properties have been identified in components such as silk^1^ and cellulose nanocrystals,^2^ contributing to their structural integrity and responsiveness to mechanical stimuli. Similarly, animals employ piezoelectricity in biological processes, with collagen in connective tissues and bones exhibiting notable piezoelectric behavior crucial for functions such as bone remodeling and repair.^3^ Proteins and their derivatives (such as glycine crystals) and peptides (such as diphenylalanine) exhibit notable piezoelectric characteristics,^4^ especially in self-assembled nanotubes.^5^ The M13 phage virus, with its helical protein-clad filamentous structure, can be used to create highly ordered crystalline structures.^6^ Ultimately, the epidermis of living human skin possesses a continual electric dipole moment oriented perpendicular to its surface, which reacts to mechanical stimuli, generating electrical signals transmitted to the neural system.^7^

The convergence of piezoelectric materials with micro- and nanotechnologies has sparked revolutions in biotechnology, bioelectronics, and healthcare. From wearable sensors^8^ for personalized health monitoring^9^ to intricate actuators orchestrating precise cellular manipulations,^10^ these innovations showcase the transformative potential of piezoelectricity in enhancing human well-being. As we delve deeper into the realms of biomedicine and healthcare, it is crucial for piezoelectric materials to prioritize biocompatibility and mechanical flexibility, often necessary for specific applications.^11,12^ Furthermore, the pervasive use of these devices demands materials that are not only eco-friendly but also amenable to large-area processing and thin-film fabrication.^13^

A common metric to quantify piezoelectricity is the *d*_33_ coefficient, which measures the amount of electric charge generated per unit of mechanical stress applied perpendicular to the electric field. A higher *d*_33_ coefficient indicates a more robust piezoelectric response, enhancing the energy conversion efficiency. While traditional materials such as lead zirconate titanate (PZT) and barium titanate (BTO) possess large piezoelectric responses (200–600 pm V^–1^),^14–16^ they are mechanically rigid,^17^ brittle,^18^ and toxic.^19,20^ Moreover, their production and disposal processes are far from being environmentally friendly.^19^ Among synthetic piezoelectric polymers, polyvinylidene fluoride (PVDF) is widely used for flexible transducers;^21–24^ however, it generates fluorine waste, leading to significant environmental concerns.^25^ Table S1 contains a list of soft synthetic piezoelectric polymers. Therefore, increasing interest has been directed toward the study of natural biobased piezoelectric materials.^26^ These materials show well-organized structures and low-symmetry patterns, enabling efficient electromechanical interactions.^8,27^ Table S2 contains a list of piezoelectric biopolymers.

Our interest has focused on chitosan, a cost-effective natural biopolymer derived from chitin, which is the second most abundant polysaccharide on Earth.^28,29^ This material offers numerous advantages such as biocompatibility,^30^ biodegradability,^31^ and the ability to easily form thin films.^29,32^ Chitosan can be extracted as a food industry byproduct from the exoskeletons of crustaceans;^33^ therefore, it is considered “sustainable by design”. In this regard, numerous studies focus on developing methods that prioritize eco-friendly practices for its acquisition and processing.^34^ These desirable properties make chitosan highly suitable for a range of industrial and biomedical applications, including tissue engineering,^35^ wound healing,^36^ and optical sensors,^37,38^ due to the transparency of the resulting films.^39^ The study of the electromechanical properties of this biopolymer started decades ago^40^ with more recent investigations focusing on thin films.^41,42^ However, due to the amorphous portion of the film matrix, the piezoelectric properties of pure chitosan appear weak.^43^ The *d*_33_ coefficient of pristine thin-film chitosan is, indeed, in the range of 5–9 pm V^–1^.^26,44^ One strategy to enhance the piezoelectric response consists of the formulation of chitosan-based composite materials.^44^ Recent studies show that increasing the polyhydroxybutyrate (PHB) content in chitosan films enhances crystallinity, and, as a consequence, the *d*_33_.^44^ Another study successfully crafted a flexible piezoelectric pressure sensor using biodegradable glycine and chitosan films, leveraging chitosan’s flexibility and glycine’s piezoelectric potential.^45^ Additionally, blending chitosan with nanocellulose structures like cellulose nanofibrils^46^ or cellulose nanocrystals^47^ hints at potential paths for enhancing the piezoelectric performance. Other examples include the use of chitin or chitosan nanofibers with poly­(vinylidene fluoride) (PVDF)^48^ and H_2_SO_4_-treated PEDOT.^10^ One breakthrough in the field occurred when *d*_33_ values up to 15 pm V^–1^ were obtained by optimizing the neutralization conditions of pristine chitosan thin films in an alkaline environment. Table S3 lists various chitosan-based materials categorized^24^ by the production methodology, the *d*_33_ measurement technique, and the values obtained.

Despite extensive research on chitosan thin films, the piezoelectricity of all-natural chitosan soft thin films has not been investigated so far. Piezoelectric materials that can elastically deform are of great interest in wearable skin-like devices for health monitoring,^49–51^ energy scavenging,^52,53^ and actuation.^52,53^ Here, we report a study of the nanoscale piezoelectricity of all-natural soft thin films prepared from chitosan, glycerol, and chitin/surface-deacetylated chitin nanocrystals. We achieved *d*_33_ values of up to 18.7 ± 1.1 pm V^–1^ for the chitosan soft thin films, an improvement of 2-fold compared to the 8.9 ± 0.6 pm V^–1^ observed in pure chitosan films produced in our laboratories. The enhanced piezoelectric response is explained by an increased crystallinity due to the addition of natural biobased chitosan/chitin NCs. Traditional methods like thermal annealing,^54^ mechanical stretching,^55^ or electrical poling^56^ enhance piezoelectricity by aligning dipoles within the material, which increases the order in crystalline regions. In contrast, our films exhibit piezoelectricity without such treatments by the incorporation of a nanostructured chitin component that enhances the piezoelectric response. The exact mechanism behind the preferential ordering and dipole alignment in our films remains unclear. Previous studies showed that nanocrystals can self-assemble into chiral nematic structures, similar to Bouligand formations in crustacean shells, where the properties of these structures are significantly dependent on the fabrication methods and experimental conditions. A comprehensive study to fully understand these mechanisms would require a systematic and extensive experimental approach, which is beyond the scope of this work.

Our accurate extraction of the *d*_33_ in soft thin films entails two critical challenges: (i) achieving high sensitivity of the piezoresponse force microscopy (PFM) transduction without damaging the surface and (ii) extracting the effective *d*_33_ that should not be affected by the electrostatic coupling between the cantilever and the surface. We have ensured proper matching of the spring constant of the cantilever with the polymer surface (essential to achieve the desired sensitivity and avoid surface indentation). Moreover, we have refined a methodology for the extraction of the *d*_33_ coefficient while removing the electrostatic contribution that may completely override the piezoelectric response, hampering the measurement.^57,58^ The calibration with a lithium niobate sample is performed at the beginning of each measurement series to determine the tip sensitivity. The content of NCs has been optimized to achieve elastic deformation up to 40% strain and a Young’s modulus of about 100 MPa, aligning closely with the physiological modulus of soft tissues. This opens up promising avenues for the construction of skin-mounted transducers, sensors, and energy scavengers that are both biocompatible and sustainable by design, as well as for the realization of green Internet of Things devices.

## Results and Discussion

### Film Preparation and Characterization

Chitin nanocrystals (ChNCs) and surface-deacetylated chitin nanocrystals (CsNCs) were synthesized as reported in the literature, with minor modifications. The details of the production process are described in the Experimental Section. The ChNCs and CsNCs were characterized from a dimensional and structural point of view. Dynamic Light Scattering (DLS) analysis highlights a length of 350 and 300 nm for ChNCs and CsNCs, respectively, and a *Z*-potential of about +55 mV for both, in agreement with literature data.^59^ Fourier Transform Infrared Spectroscopy (FT-IR) analyses, previously reported,^60,61^ do not show significant differences between the two samples, confirming that the deacetylation step does not introduce additional structural modifications. Finally, the degree of deacetylation (*D*_D_) is calculated as follows:^62^

1 $$D_{D}=100\times \left(4-0.583093\times W_{C/N}\right)$$

where *W*_C/N_ is the weight carbon-to-nitrogen ratio, as determined by CHNS analysis. ChNCs and CsNCs are found to have values of 4.6% and 36.8%, respectively. Three different film compositions were selected for the study: pure chitosan (*C*_100_) and two nanocomposite formulations containing chitosan (40 wt %), glycerol (40 wt %), and either ChNCs (20 wt %) or CsNCs (20 wt %) (C_40_G_40_ChNCs_20_ and C_40_G_40_CsNCs_20_, respectively). Films were cast on various substrates including glass and Kapton foils using an automatic applicator that allows for good uniformity and reproducibility. For a full overview of the production methods of our films, see the Experimental Section. As shown in Figure 1a, our fabrication method yields transparent (see Figure S1 for the optical absorption measurement), flexible, and stretchable thin films with thicknesses in the range of 15–20 μm. These properties are particularly attractive for applications in electronics and optoelectronics.^63^

**Figure 1:**
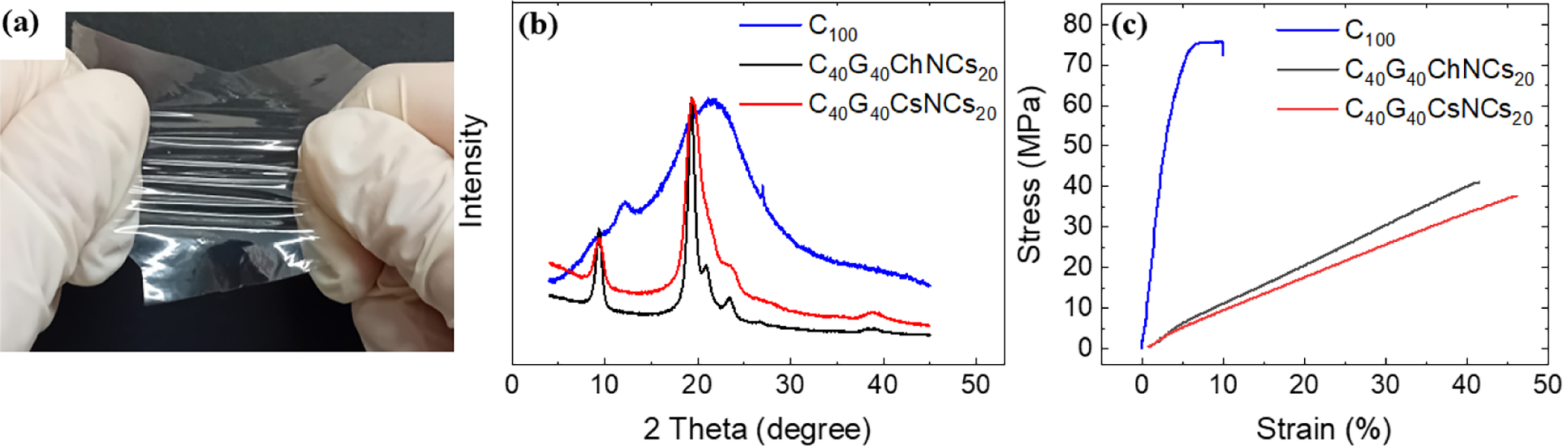
(a) Optical image of a thin film composed of chitosan, glycerol, and chitin nanocrystals, exhibiting transparency, flexibility, and stretchability. (b) XRD pattern of C_100_ (blue curve); C_40_G_40_ChNCs_20_ (black curve); and C_40_G_40_CsNCs_20_ (red curve). (c) Stress/strain curves of the former films with the same color coding.

Chitosan-based films normally possess a partially crystalline structure.^64^ The X-ray diffraction (XRD) pattern of C_40_G_40_ChNCs_20_ or C_40_G_40_CsNCs_20_ (represented by the black and red lines in Figure 1b, respectively) shows sharp and narrow diffraction peaks, indicating a high degree of crystallinity. The most notable peaks are observed at 2-theta angles of ∼9.5° and ∼21.0°, as reported in previous studies.^65,66^ We do not observe notable differences between the crystallinity of films containing ChNCs and those containing CsNCs. The partial crystallinity of neat chitosan films is instead revealed by the presence of two prominent peaks at ∼9° and ∼12°, along with a third faint peak at about ∼19°. These findings are consistent with previous reports.^24,67,68^ Additionally, we performed XRD measurements on a film composed of chitosan (50 wt %) and glycerol (50 wt %) (C_50_G_50_) to investigate the influence of glycerol on the structural properties. The diffractogram of this sample shows no crystalline peaks, revealing a completely amorphous material. The comparison between these last two diffractograms is shown in Figure S8. While it is difficult to accurately quantify the crystallinity of the films, the XRD spectra suggest that the presence of NCs in chitosan films enhances their crystallinity.

Tensile tests provide information about key mechanical parameters such as maximum load, Young’s modulus, and elongation at break (see Figure 1c). The results of the tests demonstrate that the presence of glycerol alters the stress–strain curve, causing a nonlinear behavior with large deformation at low stress and without a yield strength point.^69^ The film with pure chitosan can endure a maximum load of ∼70 MPa and ∼8% elongation before breaking. This leads to Young’s modulus of ∼875 MPa. Combining chitosan, glycerol, and NCs results in films with an excellent compromise between stiffness and deformability, with values of elongation at break, maximum load, and Young’s modulus being ∼40%, ∼40 MPa, and ∼100 MPa, respectively. These values interestingly align with those of tissues like tendons and muscles,^70^ prospecting applications in prosthetics and soft robotics.^71^ To further investigate the role of glycerol, we tested the C_50_G_50_. The results show that the presence of glycerol yields greater stretchability with respect to the film composed solely of chitosan, with a tensile strength decreasing to approximately 20 MPa, Young’s modulus reduced to around 15 MPa, and the strain at break increasing significantly to 60%. Comparing C_40_G_40_ChNCs_20_ and C_40_G_40_CsNCs_20_ with C_50_G_50_, we infer that the inclusion of NCs increases both the Young’s modulus and the tensile strength while maintaining strain values above 40%.

Additionally, to better understand the isolated mechanical behavior of NCs, we produced and tested a film composed of CsNCs (70 wt %) and the minimum amount of glycerol (30 wt %) necessary to make the film manageable (CsNCs_70_G_30_). These films exhibit a Young’s modulus similar to that of C_100_, but with a reduced tensile strength of approximately 25 MPa. This is likely due to the limited mobility of the polymer chains, restricting the material’s deformability and causing it to fracture at lower stress levels. The comparison between all samples tested is shown in Figure S7. Adjusting the percentage of NCs allows for fine-tuning of the mechanical properties for targeted applications. Besides their desirable mechanical properties, optical transparency expands the range of their potential applications to include optical sensors and transducers.

### Piezoresponse Force Microscopy (PFM) Analysis

We used an atomic force microscope (AFM) in contact mode to examine the properties of our films. Raster scans in the PFM mode were performed over selected areas of the sample surface, thus enabling simultaneous acquisition of the topography and out-of-plane PFM phase images. Using a conductive tip with a low spring constant of 0.1 N m^−1^ minimizes the risk of damaging the test material and ensures the required sensitivity for the precise electromechanical characterization of these soft materials. Analysis of the topography reveals a distinctive texture in films containing nanocrystals, unlike those composed solely of chitosan, as is evident from Figure 2a–c. The nanocrystals are uniformly distributed throughout the chitosan matrix and form intricate, intertwined patterns, which continue to be observed even in films with higher NC concentrations, as shown in Figure S3. This arrangement is clearly visible in Figure S2, where AFM images in tapping mode reveal the topography over a larger scan size of 10 × 10 μm^2^. Roughness measurements also reflect the presence of NCs (see Table S4), yielding values in the range of 3.0–5.0 nm compared to the 0.5 nm of pure chitosan (calculated over a 1 μm × 1 μm area on multiple locations of the films).

**Figure 2:**
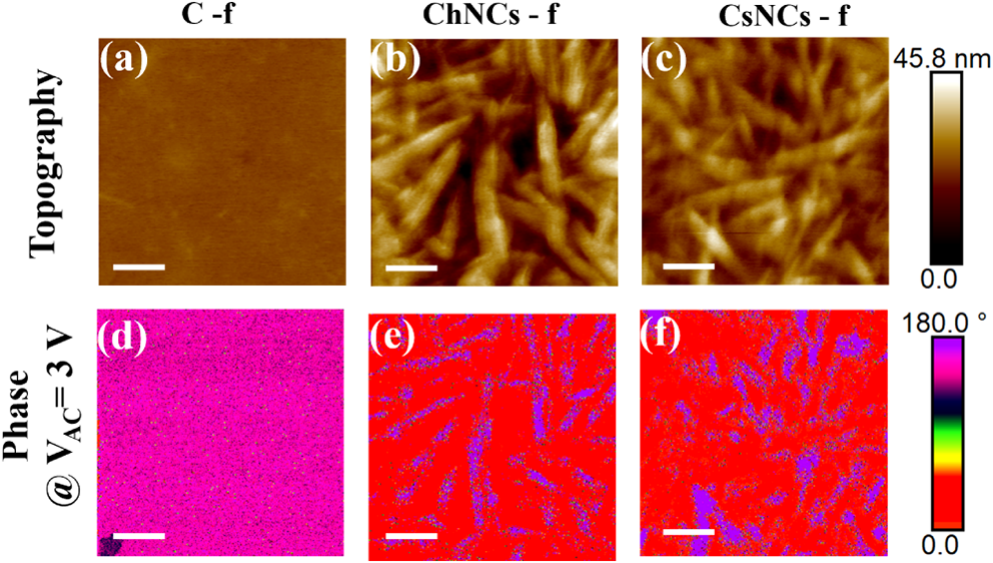
PFM images of the topography (a–c) and phase at *V*_AC_ = 3 V (d–f) of C_100_, C_40_G_40_ChNCs_20_, and C_40_G_40_CsNCs_20_. The scale bar is 200 nm in all subplots, while the color scales on the right refer to each subplot in their respective line.

Out-of-plane PFM phase imaging enables the visualization and mapping of variations in the piezoelectric response across a sample surface with high spatial resolution. This technique can reveal important insights into the electromechanical behavior of materials, such as ferroelectric/piezoelectric domains and domain-switching behavior. We used a conductive gold substrate to generate an electric field in the sample, while the conductive PFM cantilever acts as the second electrode. Figure 2d–f provides the results of out-of-plane PFM phase imaging for our films at a *V*_AC_ bias of 3 V. For all measurements, the *V*_DC_ was adjusted to approximately 0.4 V, a value closely aligned with the common surface electrostatic potential, *V*_EL_, across all three samples, as detailed in subsequent sections. This DC voltage was selected to significantly reduce electrostatic forces in the cantilever oscillation, thereby emphasizing the piezoelectric response. Under these experimental conditions, oppositely oriented vertical polar domains display an out-of-plane phase contrast of 180°. When probed using *V*_AC_ = 3 V, pure chitosan films display a uniform out-of-plane phase of oscillation across the scanned area (Figure 2 d). In contrast, films containing NCs reveal alternating regions with a 180° phase difference, indicative of piezoelectric domains. This phenomenon is particularly pronounced in areas in which nanocrystals are present within the film. Given that measurements were taken at a fixed frequency, specifically the cantilever resonance frequency measured at a single point on the sample, the observed phase variation can also be linked to changes in contact forces experienced by the cantilever due to variations in topography and contact stiffness (i.e., matrix vs nanocrystals). Additionally, the electrostatic surface potential (*V*_EL_) across the sample can vary from point to point. Since the scanning process operates at a fixed *V*_DC_ bias, this variation in *V*_EL_ cannot be accounted for during a raster scan, further influencing the reliability of phase measurements across the sample. These are also the reasons why amplitude images were excluded as they do not provide meaningful data for evaluating the piezoelectric performance of these samples.

Nonetheless, the bimodal distribution of phase, characterized by two populations separated by 180°, suggests that the findings in Figure 2e,f represent the initial evidence showcasing the piezoelectric nature of all examined films.

While PFM imaging is crucial for surface domain visualization, it lacks the precision necessary for accurate *d*_33_ coefficient quantification. Typically, surface scans incorporate an electrostatic capacitive component alongside the piezoelectric, posing challenges in its elimination during imaging. As shown in Figure 3a, the capacitor represents the capacitive coupling between the cantilever and the sample. When a sinusoidal voltage *V*_AC_ in addition to a constant voltage *V*_DC_ is applied to the substrate, displacement *Z* is recorded through cantilever deflection. The displacement *Z* depends on *V*_DC_ and *V*_AC_ through eq 2:^57^

2 $$Z=d_{33}V_{\mathrm{AC}}+\frac{1}{k}\frac{\partial C}{\partial Z}V_{\mathrm{AC}}\left|V_{\mathrm{DC}}-V_{\mathrm{EL}}\right|$$

Here, *k* is the tip spring constant of the cantilever (0.1 N m^–1^), and ∂*C*/∂*Z* is a term accounting for the tip–sample capacitive coupling, which varies with *Z*. The *Z* response is the sum of two addends: the first component is related to the piezoelectric contribution, and it is linearly dependent on *V*_AC_ and independent of *V*_DC_. The second component is an electrostatic artifact, which grows as the applied *V*_DC_ voltage moves away, i.e., the electrostatic potential *V*_EL_.^57^ Figure 3b depicts the qualitative influence of *V*_DC_ on *Z*. Point 2 is the minimum of the *Z* vs *V*_DC_ curve, which is reached at *V*_DC_ = *V*_EL_. At this point, the electrostatic coupling between the cantilever and the surface is perfectly counterbalanced by the applied *V*_DC_; therefore the displacement resulting from electrostatic forces is zero, and only the piezoelectric contribution is present. Point 1 and Point 3 are characterized by a larger electrostatic contribution compared to Point 2 (Figure 3 c–e). This is due to a larger distance between *V*_DC_ and *V*_EL_ at Points 1 and 3 compared to Point 2. In this scenario, higher *V*_AC_ leads to higher *Z* responses both at *V*_EL_ and away from it. Therefore, an accurate estimation of the *d*_33_ coefficient requires the extrapolation of the slope of the *Z* versus *V*_AC_ curve (Figure 4) at *V*_DC_ = *V*_EL_. Notably, other reported methods extract the *d*_33_ at constant |V_DC_ – V_EL_| values.^57,72^ While this approach reduces errors, it does not eliminate them entirely as ∂*C*/∂*Z* may still depend on the biasing voltage.

**Figure 3:**
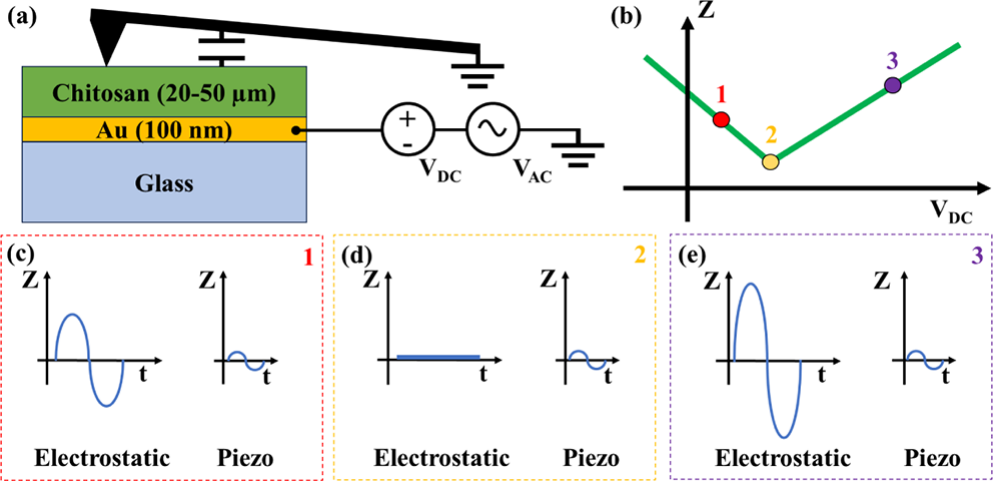
(a) Schematics showing the grounded PFM cantilever capacitively coupled to our sample with an AC and DC voltage applied to the gold electrode. (b) Displacement response of the biopolymer as a function of the applied *V*_DC_ and (c–e) unraveling of the two contributions (electrostatic and piezo) to the displacement *Z* at three representative points.

**Figure 4:**
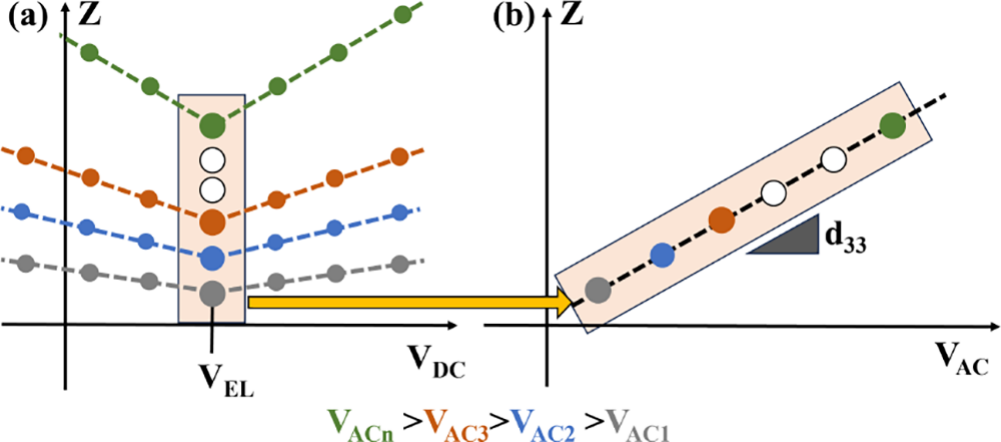
(a) Qualitative *Z* (i.e. displacement) as a function of *V*_DC_ at several *V*_ACs_ and (b) a focus on the increase of *Z* as a function of *V*_AC_ when *V*_DC_ is set to *V*_EL_. The slope of graph (b) can be used to extrapolate the piezoelectric coefficient *d*_33_ of the material through eq 2.

Using PFM, we evaluated displacement *Z* as a function of *V*_DC_ and *V*_AC_. Specifically, we swept *V*_DC_ from −1.75 to 1.75 V with 0.25 V increments, while measuring *Z*. We then repeated the sweep at several *V*_AC_ ranging from 0.5 to 3 V with 0.25 V increments. The results of our tests on C_100_, C_40_G_40_ChNCs_20_, and C_40_G_40_CsNCs_20_ are reported in Figure 5. Seen as a function of *V*_DC_, each displacement (*Z*) curve exhibits a V-shape trend. Generally, higher values of *V*_AC_ produce larger *Z*. The presence of both ChNCs and CsNCs in the films also leads to larger *Z* for all values of *V*_DC_, including *V*_DC_ = *V*_EL_. This indicates that the presence of nanocrystals induces a greater piezoelectric response.

**Figure 5:**
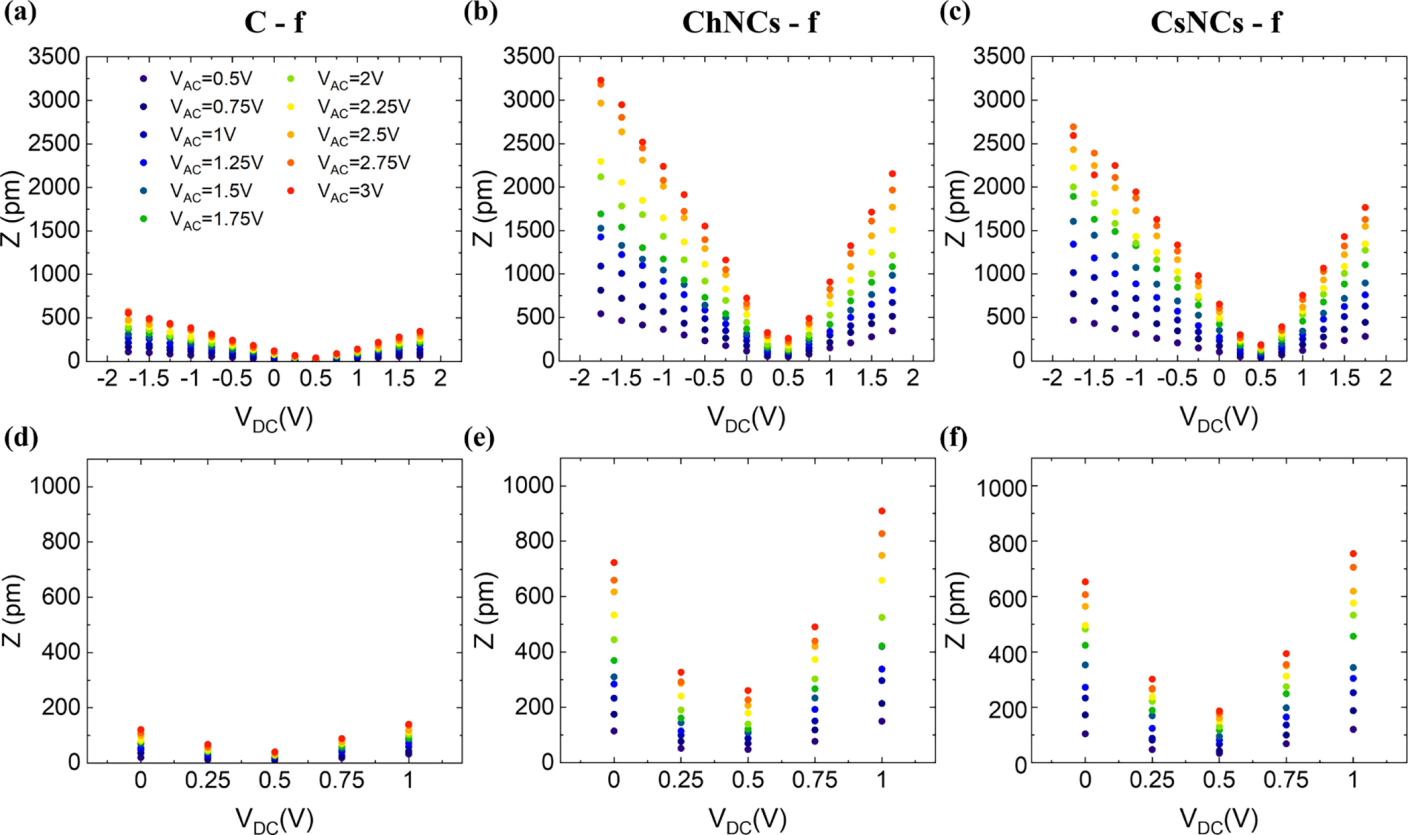
(a–c) Graphs and (d–f) close-ups showing the displacement *Z* trend as a function of *V*_DC_ (−1.75 to 1.75 V, while 0 to 1 V in the close-ups) at different *V*_AC_ (0.5 to 3 V) for C_100_ (a,d), C_40_G_40_ChNCs_20_ (b,e), and C_40_G_40_CsNCs_20_ (c,f). The plots show the data before the normalization by the quality factor (*Q*) of the cantilever resonance curve.

To correctly estimate the piezoelectric coefficient *d*_33_, the *Z* displacement values shown in Figure 5 need to be normalized by the quality factor *Q* of the cantilever, calculated from its resonance curve. This normalization also takes into account the amplification factor of the lock-in. To identify the value of *V*_EL_ at each *V*_AC_, which could fall in between two measurement points, we fit for each film the displacement *Z* as a function of *V*_DC_ and note the intersection voltage of the descending and ascending slopes as the precise value of *V*_EL_ (Figure 6). In this and the following analyses, we excluded the data points corresponding to *V*_AC_ = 2.75 V and *V*_AC_ = 3 V, as we revealed the presence of nonlinear distortion effects.

**Figure 6:**
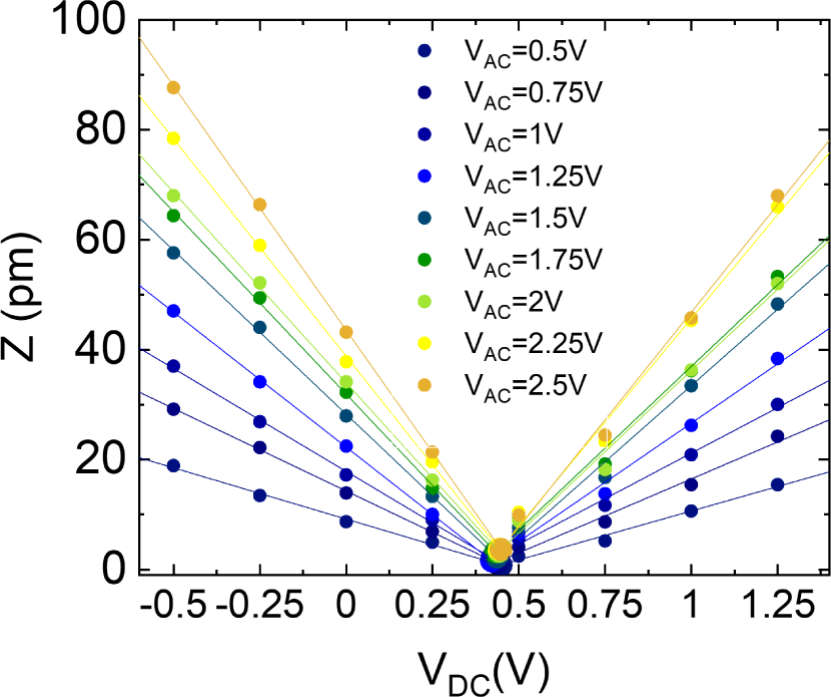
Representative fits of *V*_DC_ sweeps with two straight lines intersecting at *V*_DC_ ∼ *V*_EL_ in a C_40_G_40_CsNCs_20_. In this example, *V*_EL_ ∼ 0.4 V for all values of *V*_AC_.

We then set *V*_DC_ = *V*_EL_ and interpolate the experimental points of *Z* as a function of *V*_AC_ to extrapolate the *d*_33_. For each sample, measurements were carried out over a 2 × 2.5 cm^2^ area, selecting four points at distinct locations to ensure a representative and spatially distributed evaluation of the material’s piezoelectric response. The graphs in Figure 7 show the mean and standard deviation of all measurements for C_100_ (a), C_40_G_40_ChNCs_20_ (b), and C_40_G_40_CsNCs_20_ (c). As expected, *Z* plotted as a function of *V*_AC_ at *V*_DC_ = *V*_EL_ displays linear trends. The slope of the fitting line represents the piezoelectric coefficient *d*_33_. Each point in Figure 7 averages five different measurement points, while the error bars are calculated as half the difference between the maximum and minimum values. We estimate values of *d*_33_ of ∼8.9 ± 0.6 pm V^–1^, ∼18.7 ± 1.1 pm V^–1^, and ∼15.0 ± 0.7 pm V^–1^ for C_100_, C_40_G_40_ChNCs_20_, and C_40_G_40_CsNCs_20_, respectively. Notably, the introduction of NCs leads to a ∼2-fold enhancement in the piezoelectric coefficient value. This evidence points to points to increased crystallinity and enhanced piezoelectric properties.

**Figure 7:**
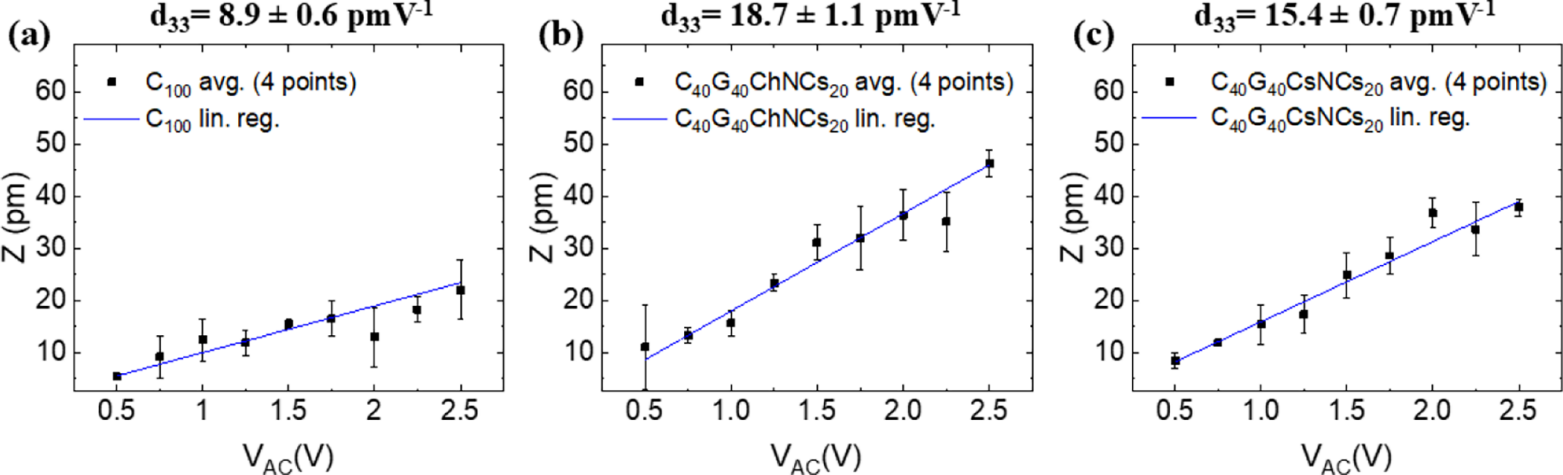
Average piezoresponse *Z* as a function of *V*_AC_ fitted linearly for C_100_ (a), C_40_G_40_ChNCs_20_ (b), and C_40_G_40_CsNCs_20_ (c). The slope of the curves, hence, *d*_33_, is printed at the top of the graphs.

To investigate how the amount of NCs affects the film properties, we produced and tested samples with varying concentrations of CsNCs, along with reference sample C_50_G_50_. The compositions tested were as follows: chitosan (47.5 wt %), glycerol (47.5 wt %), and CsNCs (5 wt %) (C_47.5_G_47.5_CsNCs_5_); chitosan (30 wt %), glycerol (30 wt %), and CsNCs (40 wt %) (C_30_G_30_CsNCs_40_). The results show that films containing 5 wt % NCs exhibit a negligible piezoelectric response, similar to C_50_G_50_, with a *d*_33_ value close to zero. This lack of piezoelectric response is attributed to a low number of NCs in the films, which are sparsely distributed in the matrix and difficult to detect by PFM. In contrast, C_30_G_30_CsNCs_40_ showed a *d*_33_ value of approximately 13 pm V^–1^, comparable to C_40_G_40_CsNCs_20_. The results of these experiments are shown in Figures S3 andS6.

We stress that the observed values for pure chitosan films are in line with those reported in previous literature reports, further validating our measurements. Although *d*_33_ values reported for pure chitosan films in the literature are comparable to our results,^24^ the key novelty of our work lies in the development of a fully biobased and sustainable soft thin film, whereas most studies focus on non-soft, non-stretchable thin films. Furthermore, this approach allows for an easy modulation of not only the piezoelectric properties but also the physical and mechanical properties of the films by simply varying the composition, with piezoelectric performance remaining robust even at higher NC content. Additionally, our study proposes a refined methodology to accurately extract the *d*_33_ by canceling the effect of capacitive coupling between the cantilever and the sample under investigation. The same methodology was also used with a sample of gold (Au) that is known to be non-piezoelectric, thus serving as a reference. Figure S4 shows the displacement values at *V*_DC_ = *V*_EL_ for Au. The *d*_33_ value extracted for Au is close to the detection limit of the PFM instrument used in this study, which is approximately 1 pm V^–1^. Au can therefore be considered non-piezoelectric, confirming the validity and effectiveness of our methodology. The calibration process also involved applying the previously described extraction method to a lithium niobate standard sample. This was done by adjusting the tip’s sensitivity to match the datasheet values of *d*_33_ for lithium niobate. The results of this procedure are shown in Figure S5.

Additional experiments were performed to investigate potential ferroelectric behavior by collecting images of double-box switching regions while applying a DC voltage of 12 V, which is the maximum voltage supported by our PFM instrument.^73^ The results did not show any detectable change in the phase signal (see Figure S9). This outcome is not surprising, considering the thickness of the chitosan films and the low *d*_33_ coefficients typically reported for similar materials, which generally require voltages exceeding 20 V to induce ferroelectric switching.^74^ Therefore, no conclusions can be drawn regarding the ferroelectricity of the chitosan films based on these measurements.

## Conclusion

This study presented an innovative approach to developing piezoelectric biosoft thin films. This was achieved by enhancing the crystallinity of chitosan thin films through the incorporation of either chitin or surface-deacetylated chitin nanocrystals into the chitosan matrix. We observed a more-than-2-fold increase in the values of the *d*_33_ coefficient (up to 18.7 pm V^–1^ compared to pure chitosan films 8.9 pm V^–1^) when chitin or surface-deacetylated chitin nanocrystals were incorporated into the films. Engineering efforts could lead to further enhancements of the crystalline structure, thereby improving the piezoelectric properties of the films. In this work, we also showed a universal method for measuring the piezoelectric properties of soft films. This was achieved by separating the piezoelectric and electrostatic responses via PFM. In addition to the nanocomposite soft films, we also examined the piezoelectric response of gold and lithium niobate using the proposed PFM method. The choice of these two reference materials, gold, which lacks a piezoelectric effect,^75^ and lithium niobate,^76^ a widely recognized piezoelectric material, further demonstrates the versatility and reliability of the technique, confirming its applicability to a broad range of materials. Additionally, the piezoelectric characteristics demonstrated by the soft thin films at the mesoscopic scale could be harnessed to generate voltage from biological deformations on larger scales. When combined with optical transparency, biocompatibility, and the capacity for large-scale production through biowaste upcycling, these findings suggest the potential of chitosan-based soft thin films for crafting sustainable-by-design transducers and sensors. This opens avenues for prosthetics, soft robotics, advanced human–machine interfaces, and applications in the Internet of Things.

## Experimental Section

### Materials

Chitosan (high MW), glycerol (MW, 92.09 g mol^–1^), chitin flakes, and acetic acid (99 wt %) were purchased from Sigma-Aldrich [CAS: 9012–76–4]. Deionized water was employed in all experiments.

### Preparation of Chitin Nanocrystals (ChNCs) and Surface-Deacetylated Nanocrystals (CsNCs)

Chitin and surface-deacetylated chitin nanocrystals were synthesized, as reported in the literature with minor modifications.^60^ Chitin flakes (purchased from Sigma-Aldrich [CAS: 1398–61–4]) (35 mg/mL) were mixed with a 3M HCl solution and constantly mixed (100 rpm) with a mechanical head stirrer for 4 h under reflux. The resulting mixture was cooled, centrifuged (4700*g*, 15 min), filtered, and rinsed in water (3×). A colloidal suspension was formed by sonication (40% amplitude for 10 min by means of a Branson Digital Sonifier equipped with a 20 kHz horn tip probe). After sonication, the mixture was diluted in water, and a 1 M NaOH solution was added to precipitate the ChNCs. The ChNCs were then centrifuged and washed with water to achieve neutral pH. The amount of ChNCs in the resulting gel was determined by drying the sample at 100 °C under vacuum for 24 h. A concentration of 11.7% was detected. To obtain the CsNCs, ChNCs (16 mg/mL) were dispersed in a 12.5 mL NaOH solution and refluxed under constant stirring (100 rpm) for 12 h. The resulting mixture was cooled, and the CsNCs were collected and purified as described above. The amount of CsNCs in the gel was determined gravimetrically as for ChNCs. A concentration of 15% was detected. To assess the stability of the nanocrystals in the liquid formulation, we conducted zeta potential analysis. Prior to the measurements, ChNCs and CsNCs were dispersed in acidified water (pH 4.0) at a concentration of 1 mg mL^–1^. The analysis yielded values of +54.1 ± 0.7 mV for ChNCs and +55.4 ± 1.1 mV for CsNCs. These results indicate that stable colloidal suspensions can be achieved, as zeta potential values within the range of ± 40 to ± 60 mV are typically associated with the prevention of aggregation and flocculation.^60^

### Preparation of Thin Films

Three different compositions were selected: pure chitosan films, nanocomposite films comprising chitosan (40 wt %), glycerol (40 wt %), and ChNCs (20 wt %), or CsNCs (20 wt %). Pure chitosan solutions were prepared by dissolving chitosan (250 mg) in 25 mL of aqueous acetic acid (AC) (1% v/v). Solutions to produce composite films were prepared by combining 200 mg of chitosan, 200 mg of glycerol, and the specific amount of either hydrated ChNCs (855 mg) or hydrated CsNCs (667 mg) required to reach a 20% concentration in 25 mL of aqueous acetic acid (AC) (1% v/v). The films were all prepared using the solvent casting method with an automatic applicator (Model: SAFA-219, S.A.M.A. Italia). The solutions were stirred, sonicated in an ultrasound bath (30 min), and then cast onto substrates placed on the chuck of the film applicator. In the case of films that underwent XRD analysis, a Kapton sheet was used as the casting substrate to facilitate the subsequent peeling of the dry film. The films analyzed with AFM/PFM were directly cast onto conductive (2.5 × 3 cm^2^) slides made of glass coated with a thin layer of gold (100 nm). In this case, the film was cast on approximately 3/4 of the entire surface of the slide, ensuring that a portion of the gold remained free and available to provide the necessary electrical contact for the PFM analysis.

### XRD Analysis

XRD patterns were recorded using an Empyrean Series 3 diffractometer (Malvern Panalytical, Malvern, United Kingdom). The configuration used was Reflection-Transmission Spinner 3.0. The intended wavelength used was Kα1 (Å): 1.540598. The X-ray tube voltage and current were 40 kV and 40 mA, respectively, with a focus of 12 mm in length and 0.4 mm in width. The scan analysis was performed in continuous mode in the range [5°–40°].

### Tensile Tests

Stress–strain analysis was carried out on rectangular specimens prepared using an automatic cutter by means of an MTS Insight machine equipped with a 100 N load cell. Gauge length and sample width of 20 and 10 mm were respectively used, and the crosshead speed was set to 0.5 mm/s. The Young’s modulus (*E*) was calculated from the first linear segment of the curve.

### PFM Analysis

PFM measurements were conducted using a Bruker Dimension Icon instrument (Bruker Corporation, Billerica, Massachusetts, U.S.A.) equipped with a platinum–iridium-coated conductive silicon probe (SCM-PIC-V2, Bruker Corporation, Billerica, Massachusetts, U.S.A.). The probe had a nominal spring constant *k* of 0.1 N/m, a nominal length of 450 μm, and a free resonance frequency of approximately 10 kHz. It was operated in contact mode at 40 kHz, which is the resonance frequency of the tip when in contact with the surface. An alternating voltage of 3 V was applied to the sample during scanning to capture the amplitude and phase images. The piezoelectric coefficient was determined by calibrating the tip with a lithium niobate sample. More specifically, the calibration process consisted of applying the extraction method described below to a standard sample by calibrating the sensitivity of the tip against the datasheet values of *d*_33_ lithium niobate. The results of this operation are shown in Figure S5. *Details of the d*_33_ *extraction method:* four points have been measured for each sample. Each point has been biased at various V_DC_ and V_AC_ values following these steps:

- The displacement versus frequency curve *Z*–*f* is registered for each *V*_DC_ in the range [−1.75, 1.75] V at 0.25 V step (11 *V*_DC_ values) and the maximum of the *Z*–*f* curve *max­{Z–f}* and the quality factor has been extracted after interpolation;
- The *max­{Z*–*f}* values are plotted against the corresponding *V*_DC_ after being normalized for an average quality factor *Q*;
- Points (i) and (ii) are repeated for the various *V*_AC_ in the range 0.5 V–3 V with a step of 0.25 V;
- The electrostatic voltage *V*_EL_ and the corresponding *Z* are extracted from the linear fitting of the *Z* versus *V*_DC_ for each *V*_AC_. Here, *V*_AC_ = 2.75 and 3 V are neglected because of nonlinear effects (see eq 1);
- The *Z* values extracted at Point (iv) are plotted against *V*_AC_.
- The procedure i-v is repeated for each point (4 points for each kind of film) and are linearly interpolated to find the *d*_33_ coefficient (slope for the fitting line).

## Supplementary Material





## Notes

Measurements of optical absorption of chitosan films; atomic force microscopy (AFM) analysis of chitosan-based films; piezo force microscopy (PFM) analysis; measurements and *d*_33_ extraction of gold sample; measurements and *d*_33_ extraction of lithium niobate sample; *d*_33_ extraction for films with different amount of CsNCs in chitosan/glycerol matrix; stress–strain curves for different composition of films; manufacturing technology, measuring piezoelectric technique, and piezoelectric coefficient *d*_33_ values for different soft polymers (Table 1); manufacturing technology, measuring piezoelectric technique, and piezoelectric coefficient *d*_33_ values for different biopolymers (Table 2); manufacturing technology, measuring the *d*_33_ technique and piezoelectric coefficient *d*_33_ values for different chitosan-based materials (Table 3); roughness measurements obtained from PFM scans (Table 4) (PDF)
The applications files also include the raw data of one point for each film (C_100_, C_40_G_40_ChNCs_20_, and C_40_G_40_CsNCs_20_) and the python code for the extraction of the *d*_33_ (ZIP)

## Abbreviations

PFM: Piezoresponse force microscopy
ChNCs: Chitin nanocrystals
CsNCs: Surface-deacetylated chitin nanocrystals
C_100_: Pure chitosan film
C_50_ G_50_: Chitosan (50 wt %)/Glycerol (50 wt %) film
C_40_ G_40_ChNCs_20_: Chitosan (40 wt %)/Glycerol (40 wt %)/ChNCs (20 wt %) film
C_40_ G_40_CsNCs_20_: Chitosan (40 wt %)/Glycerol (40 wt %)/CsNCs (20 wt %) film
C_47.5_ G_47.5_ChNCs_5_: Chitosan (47.5 wt %)/Glycerol (47.5 wt %)/ChNCs (5 wt %) film
C_47.5_ G_47.5_CsNCs_5_: Chitosan (47.5 wt %)/Glycerol (47.5 wt %)/CsNCs (5 wt %) film
C_30_ G_30_ChNCs_40_: Chitosan (30 wt %)/Glycerol (30 wt %)/ChNCs (40 wt %) film
C_30_ G_30_CsNCs_40_: Chitosan (30 wt %)/Glycerol (30 wt %)/CsNCs (40 wt %) film.

## References

[1] Joseph J., Singh S. G., Vanjari S. R. K. (2018). Piezoelectric
Micromachined Ultrasonic
Transducer Using Silk Piezoelectric Thin Film. IEEE Electron Device Lett..

[2] Sun B., Chao D., Wang C. (2022). Piezoelectric
Nanogenerator Based
on Electrospun Cellulose Acetate/Nanocellulose Crystal Composite Membranes
for Energy Harvesting Application. Chem. Res.
Chin. Univ..

[3] Sun Y., Zeng K., Li T. (2020). Piezo-/Ferroelectric Phenomena in
Biomaterials: A Brief Review of Recent Progress and Perspectives. Sci. China Phys. Mech. Astron..

[4] Guerin S., Stapleton A., Chovan D., Mouras R., Gleeson M., McKeown C., Noor M. R., Silien C., Rhen F. M. F., Kholkin A. L., Liu N., Soulimane T., Tofail S. A. M., Thompson D. (2018). Control of Piezoelectricity
in Amino
Acids by Supramolecular Packing. Nat. Mater..

[5] Kholkin A., Amdursky N., Bdikin I., Gazit E., Rosenman G. (2010). Strong Piezoelectricity
in Bioinspired Peptide Nanotubes. ACS Nano.

[6] Shin D.-M., Han H. J., Kim W.-G., Kim E., Kim C., Hong S. W., Kim H. K., Oh J.-W., Hwang Y.-H. (2015). Bioinspired
Piezoelectric Nanogenerators Based on Vertically Aligned Phage Nanopillars. Energy Environ. Sci..

[7] Athenstaedt H., Claussen H., Schaper D. (1982). Epidermis
of Human Skin: Pyroelectric
and Piezoelectric Sensor Layer. Science.

[8] Ali M., Bathaei M. J., Istif E., Karimi S. N. H., Beker L. (2023). Biodegradable
Piezoelectric Polymers: Recent Advancements in Materials and Applications. Adv. Healthcare Mater..

[9] Sultana A., Ghosh S. K., Sencadas V., Zheng T., Higgins M. J., Middya T. R., Mandal D. (2017). Human Skin Interactive Self-Powered
Wearable Piezoelectric Bio-e-Skin by Electrospun Poly- l -Lactic
Acid Nanofibers for Non-Invasive Physiological Signal Monitoring. J. Mater. Chem. B.

[10] Du L., Li T., Jin F., Wang Y., Li R., Zheng J., Wang T., Feng Z.-Q. (2020). Design of High Conductive and Piezoelectric
Poly (3,4-Ethylenedioxythiophene)/Chitosan Nanofibers for Enhancing
Cellular Electrical Stimulation. J. Colloid
Interface Sci..

[11] Kamel N. A. (2022). Bio-piezoelectricity:
Fundamentals and applications in tissue engineering and regenerative
medicine. Biophys. Rev..

[12] Wu Y., Ma Y., Zheng H., Ramakrishna S. (2021). Piezoelectric
Materials for Flexible
and Wearable Electronics: A Review. Mater. Des..

[13] Lemaire E., Ayela C., Atli A. (2018). Eco-Friendly
Materials for Large
Area Piezoelectronics: Self-Oriented Rochelle Salt in Wood. Smart Mater. Struct..

[14] Karaki T., Yan K., Miyamoto T., Adachi M. (2007). Lead-Free
Piezoelectric Ceramics
with Large Dielectric and Piezoelectric Constants Manufactured from
BaTiO3 Nano-Powder. Jpn. J. Appl. Phys..

[15] Smith G. L., Pulskamp J. S., Sanchez L. M., Potrepka D. M., Proie R. M., Ivanov T. G., Rudy R. Q., Nothwang W. D., Bedair S. S., Meyer C. D., Polcawich R. G. (2012). PZT-Based
Piezoelectric MEMS Technology. J. Am. Ceram.
Soc..

[16] Gao J., Xue D., Liu W., Zhou C., Ren X. (2017). Recent Progress on
BaTiO3-Based Piezoelectric Ceramics for Actuator Applications. Actuators.

[17] Fan J., Stoll W. A., Lynch C. S. (1999). Nonlinear Constitutive Behavior of
Soft and Hard PZT: Experiments and Modeling. Acta Mater..

[18] van
den Ende D. A., de Almeida P., van der Zwaag S. (2007). Piezoelectric
and Mechanical Properties of Novel Composites of PZT and a Liquid
Crystalline Thermosetting Resin. J. Mater. Sci..

[19] Panda P. K. (2009). Review:
Environmental Friendly Lead-Free Piezoelectric Materials. J. Mater. Sci..

[20] Ahamed M., Akhtar M. J., Khan M. A. M., Alhadlaq H. A., Alshamsan A. (2020). Barium Titanate
(BaTiO3) Nanoparticles Exert Cytotoxicity through Oxidative Stress
in Human Lung Carcinoma (A549) Cells. Nanomaterials.

[21] Guan Y., Tu L., Ren K., Kang X., Tian Y., Deng W., Yu P., Ning C., Fu R., Tan G., Zhou L. S. S.-E. (2023). All-Polymer
Piezoelectric Elastomer for Artificial Electronic Skin. ACS Appl. Mater. Interfaces.

[22] Stiubianu G.-T., Bele A., Bargan A., Potolinca V. O., Asandulesa M., Tugui C., Tiron V., Hamciuc C., Dascalu M., Cazacu M. (2022). All-Polymer Piezo-Composites
for
Scalable Energy Harvesting and Sensing Devices. Molecules.

[23] Tugui C., Bele A., Tiron V., Hamciuc E., Varganici C. D., Cazacu M. (2017). Dielectric Elastomers with Dual Piezo-Electrostatic
Response Optimized through Chemical Design for Electromechanical Transducers. J. Mater. Chem. C.

[24] De
Marzo G., Mastronardi V. M., Algieri L., Vergari F., Pisano F., Fachechi L., Marras S., Natta L., Spagnolo B., Brunetti V., Rizzi F., Pisanello F., De Vittorio M. (2023). Sustainable, Flexible, and Biocompatible Enhanced Piezoelectric
Chitosan Thin Film for Compliant Piezosensors for Human Health. Adv. Electron. Mater..

[25] Veiga A. G., Dias F. G. D. A., Batista L. D. N., Rocco M. L. M., Costa M. F. (2020). Reprocessed
Poly­(Vinylidene Fluoride): A Comparative Approach for Mechanical Recycling
Purposes. Mater. Today Commun..

[26] Hänninen A., Sarlin E., Lyyra I., Salpavaara T., Kellomäki M., Tuukkanen S. (2018). Nanocellulose
and Chitosan Based
Films as Low Cost, Green Piezoelectric Materials. Carbohydr. Polym..

[27] Kumar R., Bera S. (2024). Recent Approaches in Development
of Bio-Based Artificial Piezoelectric
Constructs for Biomedical Applications. Giant.

[28] Maschmeyer T., Luque R., Selva M. (2020). Upgrading
of Marine (Fish and Crustaceans)
Biowaste for High Added-Value Molecules and Bio­(Nano)-Materials. Chem. Soc. Rev..

[29] Xu C., Nasrollahzadeh M., Selva M., Issaabadi Z., Luque R. (2019). Waste-to-Wealth: Biowaste
Valorization into Valuable Bio­(Nano)­Materials. Chem. Soc. Rev..

[30] Rodrigues S., Dionísio M., López C. R., Grenha A. (2012). Biocompatibility of
Chitosan Carriers with Application in Drug Delivery. J. Funct. Biomater..

[31] Wrońska N., Katir N., Nowak-Lange M., El Kadib A., Lisowska K. (2023). Biodegradable
Chitosan-Based Films as an Alternative to Plastic Packaging. Foods.

[32] Pavinatto F. J., Caseli L., Oliveira O. N. (2010). Chitosan in Nanostructured
Thin Films. Biomacromolecules.

[33] Hamed I., Özogul F., Regenstein J. M. (2016). Industrial Applications of Crustacean
By-Products (Chitin, Chitosan, and Chitooligosaccharides): A Review. Trends Food Sci. Technol..

[34] Shamshina J. L., Barber P. S., Gurau G., Griggs C. S., Rogers R. D. (2016). Pulping
of Crustacean Waste Using Ionic Liquids: To Extract or Not To Extract. ACS Sustainable Chem. Eng..

[35] Sultankulov B., Berillo D., Sultankulova K., Tokay T., Saparov A. (2019). Progress in
the Development of Chitosan-Based Biomaterials for Tissue Engineering
and Regenerative Medicine. Biomolecules.

[36] Chen Y., Ye M., Song L., Zhang J., Yang Y., Luo S., Lin M., Zhang Q., Li S., Zhou Y., Chen A., An Y., Huang W., Xuan T., Gu Y., He H., Wu J., Li X. (2020). Piezoelectric and Photothermal Dual Functional Film
for Enhanced Dermal Wound Regeneration via Upregulation of Hsp90 and
HIF-1α. Appl. Mater. Today.

[37] Fen Y. W., Yunus W. M. M. (2013). Utilization of
Chitosan-Based Sensor Thin Films for
the Detection of Lead Ion by Surface Plasmon Resonance Optical Sensor. IEEE Sensors J..

[38] Lin S., Chang C.-C., Lin C.-W. (2012). A REVERSIBLE
OPTICAL SENSOR BASED
ON CHITOSAN FILM FOR THE SELECTIVE DETECTION OF COPPER IONS. Biomed. Eng. Appl. Basis Commun..

[39] Park J., Park B., Kim T. Y., Jung S., Choi W. J., Ahn J., Yoon D. H., Kim J., Jeon S., Lee D., Yong U., Jang J., Kim W. J., Kim H. K., Jeong U., Kim H. H., Kim C. (2021). Quadruple Ultrasound,
Photoacoustic, Optical Coherence, and Fluorescence Fusion Imaging
with a Transparent Ultrasound Transducer. Proc.
Natl. Acad. Sci. U. S. A..

[40] Fukada E., Sasaki S. (1975). Piezoelectricity of
α-Chitin. J. Polym. Sci., Polym. Phys.
Ed..

[41] Ahmad F. B., Maziati Akmal M. H., Amran A., Hasni M. H. (2020). Characterization
of Chitosan from Extracted Fungal Biomass for Piezoelectric Application. IOP Conf Ser. Mater. Sci. Eng..

[42] Praveen E., Murugan S., Jayakumar K. (2017). Investigations
on the Existence of
Piezoelectric Property of a Bio-Polymer – Chitosan and Its
Application in Vibration Sensors. RSC Adv..

[43] Hänninen A., Rajala S., Salpavaara T., Kellomäki M., Tuukkanen S. (2016). Piezoelectric
Sensitivity of a Layered Film of Chitosan
and Cellulose Nanocrystals. Procedia Eng..

[44] Toalá C. U., Prokhorov E., Barcenas G. L., Landaverde M. A. H., Limón J. M. Y., Gervacio-Arciniega J. J., De Fuentes O. A., Tapia A. M. G. (2023). Electrostrictive and Piezoelectrical
Properties of Chitosan-Poly­(3-Hydroxybutyrate) Blend Films. Int. J. Biol. Macromol..

[45] Hosseini E. S., Manjakkal L., Shakthivel D., Dahiya R. (2020). Glycine–Chitosan-Based
Flexible Biodegradable Piezoelectric Pressure Sensor. ACS Appl. Mater. Interfaces.

[46] Qin L., Zhang Y., Fan Y., Li L. (2023). Cellulose Nanofibril
Reinforced Functional Chitosan Biocomposite Films. Polym. Test..

[47] Yadav M., Behera K., Chang Y.-H., Chiu F.-C. (2020). Cellulose Nanocrystal
Reinforced Chitosan Based UV Barrier Composite Films for Sustainable
Packaging. Polymers.

[48] Hoque N. A., Thakur P., Biswas P., Saikh M., Roy S., Bagchi B., Das S., Ray P. P. (2018). Biowaste Crab Shell-Extracted
Chitin Nanofiber-Based Superior Piezoelectric Nanogenerator. J. Mater. Chem. A.

[49] Hu H., Ma Y., Gao X., Song D., Li M., Huang H., Qian X., Wu R., Shi K., Ding H., Lin M., Chen X., Zhao W., Qi B., Zhou S., Chen R., Gu Y., Chen Y., Lei Y., Wang C., Wang C., Tong Y., Cui H., Abdal A., Zhu Y., Tian X., Chen Z., Lu C., Yang X., Mu J., Lou Z., Eghtedari M., Zhou Q., Oberai A., Xu S. (2023). Stretchable Ultrasonic
Arrays for the Three-Dimensional Mapping of the Modulus of Deep Tissue. Nat. Biomed. Eng..

[50] Shu S., An J., Chen P., Liu D., Wang Z., Li C., Zhang S., Liu Y., Luo J., Zu L. (2021). Active-Sensing Epidermal Stretchable Bioelectronic
Patch for Noninvasive,
Conformal, and Wireless Tendon Monitoring. Research.

[51] Sun T., Tasnim F., McIntosh R. T., Amiri N., Solav D., Anbarani M. T., Sadat D., Zhang L., Gu Y., Karami M. A., Dagdeviren C. (2020). Decoding of Facial Strains via Conformable
Piezoelectric Interfaces. Nat. Biomed. Eng..

[52] Dagdeviren C., Joe P., Tuzman O. L., Park K.-I., Lee K. J., Shi Y., Huang Y., Rogers J. A. (2016). Recent Progress in Flexible and Stretchable
Piezoelectric Devices for Mechanical Energy Harvesting, Sensing and
Actuation. Extreme Mech. Lett..

[53] Du S., Zhou N., Gao Y., Xie G., Du H., Jiang H., Zhang L., Tao J., Zhu J. (2020). Bioinspired
Hybrid Patches with Self-Adhesive Hydrogel and Piezoelectric Nanogenerator
for Promoting Skin Wound Healing. Nano Res..

[54] Zhang M., Liu C., Li B., Shen Y., Wang H., Ji K., Mao X., Wei L., Sun R., Zhou F. (2023). Electrospun PVDF-Based
Piezoelectric Nanofibers: Materials, Structures, and Applications. Nanoscale Adv..

[55] Wang X., Xiang X., Xie J., Zhao G., Li Z., Sun X. (2024). Unleashing the Potential:
Strategies for Enhancing Performance of
Electrospun PVDF-Based Piezoelectric Nanofibrous Membranes. Fibers Polym..

[56] Megdich A., Habibi M., Laperrière L. (2024). Modeling and
Optimization of Piezoelectric
and Dielectric Properties of Poled PVDF/BT Nanocomposites. Polym. Bull..

[57] Miller N. C., Grimm H. M., Horne W. S., Hutchison G. R. (2019). Accurate
Electromechanical Characterization of Soft Molecular Monolayers Using
Piezo Force Microscopy. Nanoscale Adv..

[58] Gervacio-Arciniega J. J., Murillo-Bracamontes E. A., Toledo-Solano M., Fuentes J., Portelles J., Cruz-Valeriano E., Palomino-Ovando M. A., Ramirez-Sarabia J. A., Hernandez-Gonzalez L., Cruz M. P. (2020). Discrimination of a Ferroelectric from a Non-Ferroelectric
Response in PFM by Phase Analyses at the Harmonics of the Applied
Vac. J. Appl. Phys..

[59] Pereira A. G. B., Muniz E. C., Hsieh Y.-L. (2015). 1H NMR
and 1H–13C HSQC Surface
Characterization of Chitosan–Chitin Sheath-Core Nanowhiskers. Carbohydr. Polym..

[60] Massari D., Sgarzi M., Gigli M., Crestini C. (2024). Introducing
Hydrogen
Bond Networks in the Self-Assembly of Chitin Nanocrystals: Strong
and Flexible Bioactive Films Containing Natural Polyphenols. Adv. Sustainable Syst..

[61] Segato J., Calmanti R., Gnoato G., Cavarzerani E., Rizzolio F., Crestini C., Perosa A., Gigli M., Selva M. (2024). Fish Scales for Wearable Patches: Tailoring Films Assembled from
Fish Waste Gelatin, Carbon Dots and Chitin Nanocrystals. Adv. Sustainable Syst..

[62] dos
Santos Z. M., Caroni A. L. P. F., Pereira M. R., da Silva D. R., Fonseca J. L. C. (2009). Determination of Deacetylation Degree of Chitosan:
A Comparison between Conductometric Titration and CHN Elemental Analysis. Carbohydr. Res..

[63] Trung T. Q., Lee N.-E. (2017). Materials and Devices for Transparent
Stretchable Electronics. J. Mater. Chem. C.

[64] Ioelovich M. (2016). Crystallinity
and Hydrophility of Chitin and Chitosan. Res.
Rev.: J. Chem..

[65] Robles E., Salaberria A. M., Herrera R., Fernandes S. C. M., Labidi J. (2016). Self-Bonded Composite
Films Based on Cellulose Nanofibers
and Chitin Nanocrystals as Antifungal Materials. Carbohydr. Polym..

[66] Goodrich J. D., Winter W. (2007). T. α-Chitin Nanocrystals Prepared
from Shrimp
Shells and Their Specific Surface Area Measurement. Biomacromolecules.

[67] Fernández-Marín R., Morales A., Erdocia X., Iturrondobeitia M., Labidi J., Lizundia E. (2024). Chitosan–Chitin Nanocrystal
Films from Lobster and Spider Crab: Properties and Environmental Sustainability. ACS Sustainable Chem. Eng..

[68] Isobe N., Kaku Y., Okada S., Kawada S., Tanaka K., Fujiwara Y., Nakajima R., Bissessur D., Chen C. (2022). Identification of Chitin Allomorphs
in Poorly Crystalline Samples
Based on the Complexation with Ethylenediamine. Biomacromolecules.

[69] Callister, W. D., Jr. ; Rethwisch, D. G. Fundamentals of Materials Science and Engineering: An Integrated Approach; Wiley, 2012.

[70] McKee C. T., Last J. A., Russell P., Murphy C. J. (2011). Indentation
Versus
Tensile Measurements of Young’s Modulus for Soft Biological
Tissues. Tissue Eng., Part B.

[71] Ashuri T., Armani A., Jalilzadeh
Hamidi R., Reasnor T., Ahmadi S., Iqbal K. (2020). Biomedical
Soft Robots: Current Status and Perspective. Biomed. Eng. Lett..

[72] Algieri L., Todaro M. T., Guido F., Mastronardi V., Desmaële D., Qualtieri A., Giannini C., Sibillano T., De Vittorio M. (2018). Flexible Piezoelectric
Energy-Harvesting Exploiting
Biocompatible AlN Thin Films Grown onto Spin-Coated Polyimide Layers. ACS Appl. Energy Mater..

[73] Kim K.-E., Jeong S., Chu K., Lee J. H., Kim G.-Y., Xue F., Koo T. Y., Chen L.-Q., Choi S.-Y., Ramesh R., Yang C.-H. (2018). Configurable
Topological Textures in Strain Graded
Ferroelectric Nanoplates. Nat. Commun..

[74] Kim K., Ha M., Choi B., Joo S. H., Kang H. S., Park J. H., Gu B., Park C., Park C., Kim J., Kwak S. K., Ko H., Jin J., Kang S. J. B. (2018). Electro-Active Chitin Nanofiber Films
for Flexible Piezoelectric Transducers. Nano
Energy.

[75] Sekhar M. C., Veena E., Kumar N. S., Naidu K. C. B., Mallikarjuna A., Basha D. B. (2023). A Review on Piezoelectric Materials and Their Applications. Cryst. Res. Technol..

[76] Yue W., Yi-Jian J. (2003). Crystal Orientation Dependence of Piezoelectric Properties
in LiNbO3 and LiTaO3. Opt. Mater..

