# Supplementary material for: Enhanced Piezoelectricity in Sustainable-By-Design Chitosan Nanocomposite Soft Thin Films for Green Sensors
Source: ACS Nano. 2025 Aug 18;19(40):35322–32. doi: 10.1021/acsnano.4c12855 (PMC12530049; doi:10.1021/acsnano.4c12855)
Supplement: Supplementary file 1 [file nn4c12855_si_001.pdf]

Supporting Information

Enhanced Piezoelectricity in Sustainable-by-design Chitosan Nanocomposite Soft Thin Films for Green Sensors

*Jacopo Nicoletti, Leonardo Puppulin, Julie Routurier, Saimir Frroku, Nouha Loudhaief, Claudia Crestini, Alvise Perosa, Maurizio Selva, Matteo Gigli, Michele Back, Pietro Riello, Domenico De Fazio, Giovanni Antonio Salvatore**

J. Nicoletti[1], L. Puppulin[1], J. Routurier[2], S. Frroku[1], N. Loudhaief[1], C. Crestini[1], A. Perosa[1], M. Selva[1], M. Gigli[1], M. Back[1], P. Riello[1], D. De Fazio[1], G. A. Salvatore[1]

[1]Ca' Foscari University of Venice, Department of Molecular Science and Nanosystems, Via Torino 155, 30172 Venezia, Italy

[2] Université de Haute-Alsace (UHA), Ecole de Chimie de Mulhouse (ENSCMu), 3 Rue Alfred Werner 68200 Mulhouse, France

E-mail: giovanni.salvatore@unive.it, matteo.gigli@unive.it, leonardo.puppulin@unive.it

Measurements of optical absorption of neat chitosan films

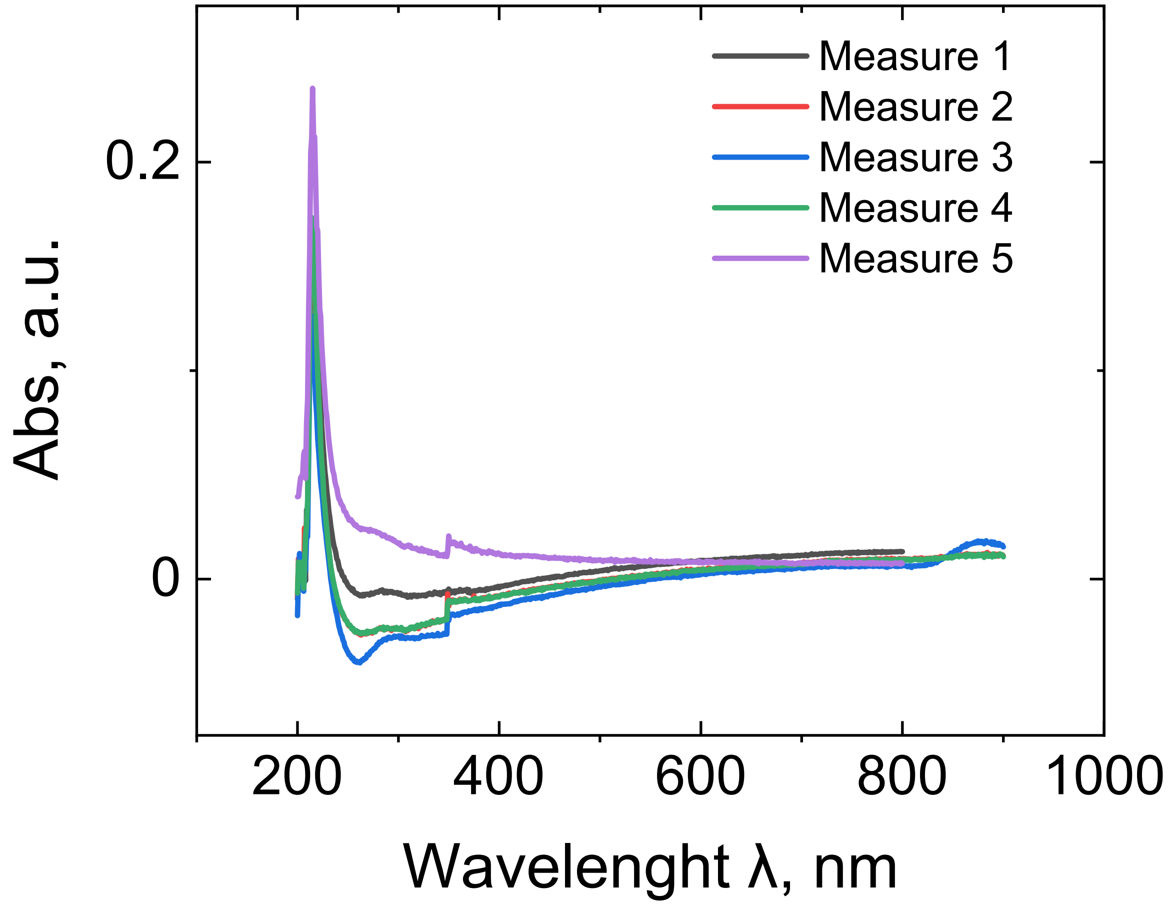

Figure S1. Optical absorption of neat chitosan films. The graph refers to five repeated measurements of the same sample. All curves appear flat in the visible range (wavelength between 400 - 800 nm) and with a constant value of approximately zero. It is also possible to observe an absorption peak around 200 nm, which is consistent with other results from studies on chitosan films in the literature. [1–3]

<u>Methodology for measurements of the optical absorption:</u> the tests were carried out on chitosan films produced by a solvent casting process (starting from 1 ml of aqueous acetic acid (AC) (1% v/v) solution with a chitosan concentration of 25 mg/ml) using an applicator film by casting the solution directly onto the sample holder. The resulting film is approximately 20 micromerters thick. The UV-Vis Spectrophotometry tests were carried out with an Agilent Cary 100 UV/Vis Spectrophotometer. The calibration process was carried out using the blank sample holder as a reference, i.e. without the film deposited on it.

Atomic Force Microscopy (AFM) analysis

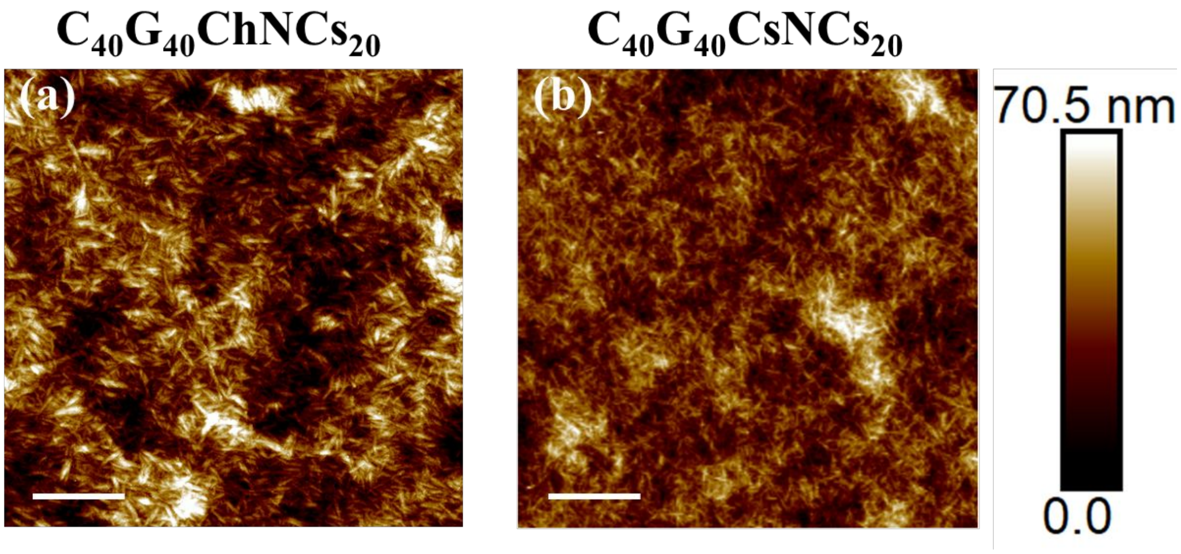

Figure S2. AFM images of topography of: $C_{40}G_{40}ChNCs_{20}$ (a), $C_{40}G_{40}CsNCs_{20}$ (b). The scan size is 10 x 10 μm^2 and the scale bar is 2 μm in all plots, while the color scales on the right refer to both plots. The measurements were conducted using a Bruker Dimension Icon instrument (Bruker Corporation, Billerica, Massachusetts, U.S.A) equipped with a silicon tip on silicon nitride cantilever (SCM-PIC-V2, Bruker Corporation, Billerica, Massachusetts, U.S.A). The probe had a nominal spring constant k of 0.4 N/m, a nominal length of 115 μm, and a free resonance frequency of approximately 70 KHz. It was operated in tapping mode.

Piezo Force Microscopy (PFM) analysis

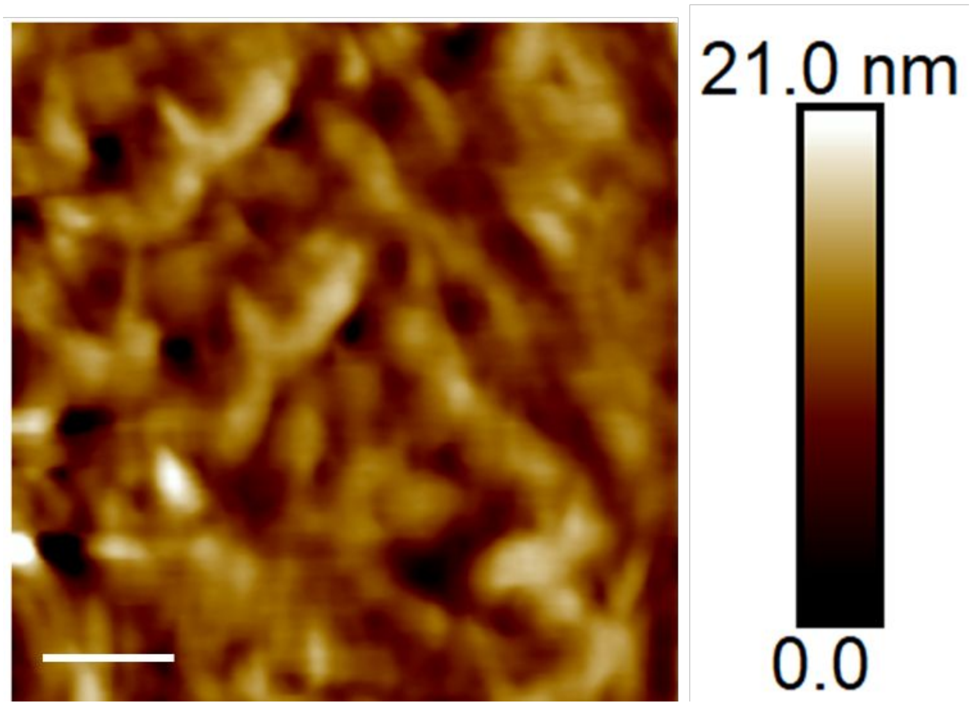

Figure S3. PFM images of the topography for $C_{30}G_{30}CsNCs_{40}$. The scalebar is 200 nm.

Measurements and d_{33} extraction of gold sample

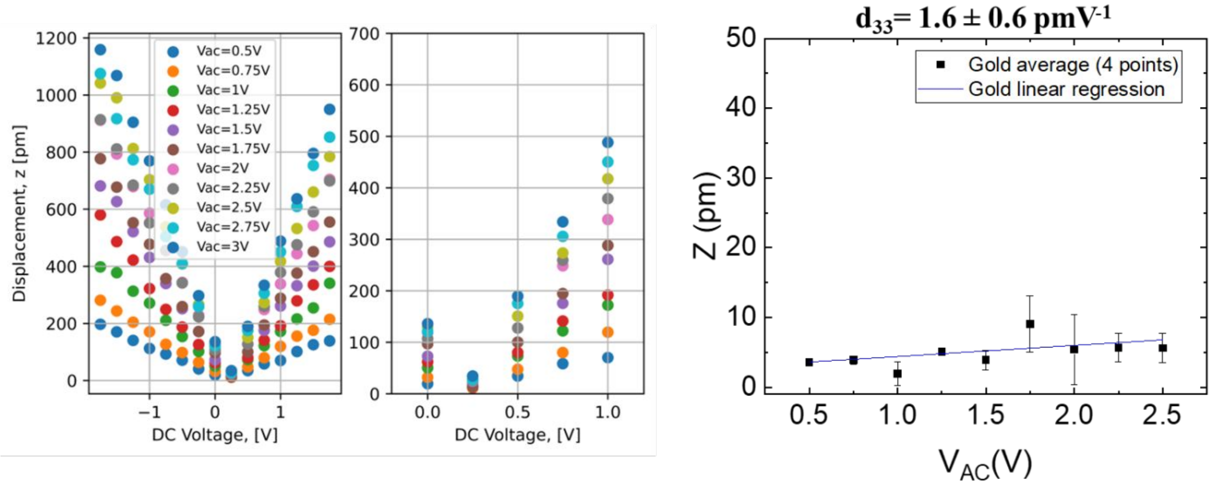

Figure S4. PFM Analysis of a gold sample: on the left, the displacement as function of V_{DC} for various V_{AC}. The experiments followed the methodology described in "Methods and Materials" and applied for the analysis of the chitosan-based films. The analysis shows a d_{33} that is about 1.6 pmV^{-1} (on the right) which is close to the limit of detection of the PFM instrument. The results confirm the non-piezoelectricity of Au and the effectiveness of our methodology.

Measurements and d_{33} extraction of Lithium Niobate sample

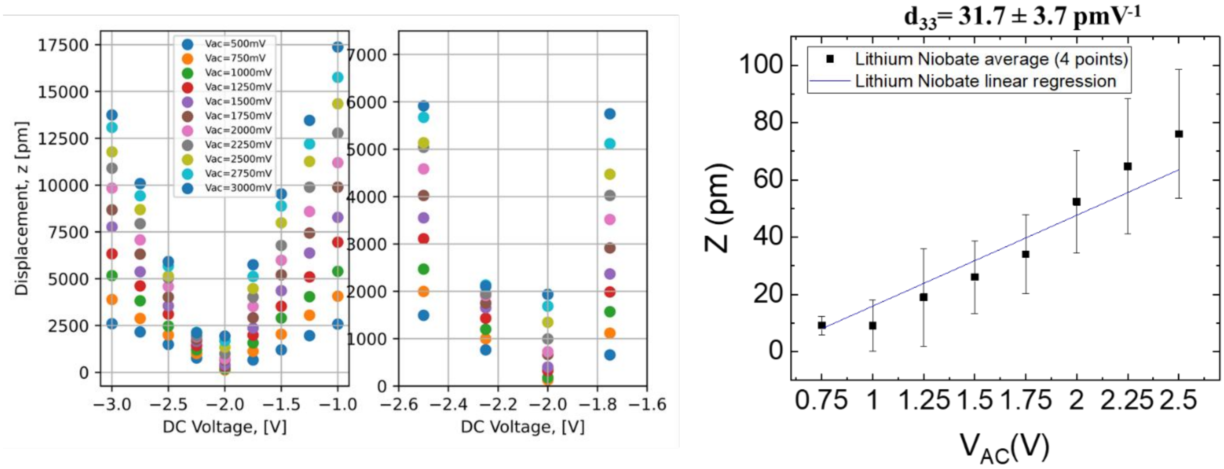

Figure S5. PFM Analysis of a Lithium Niobate sample: on the left displacement as function of V_{DC} for various V_{AC}. The measurements on the Lithium Niobate are used to calibrate the sensitivity of the tip by matching the found experimental value of d_{33} with the one of the datasheet. On the right the graph shows the value of the piezoelectric coefficient.

d_{33} extraction for films with different amount of CsNCs in chitosan/glycerol matrix

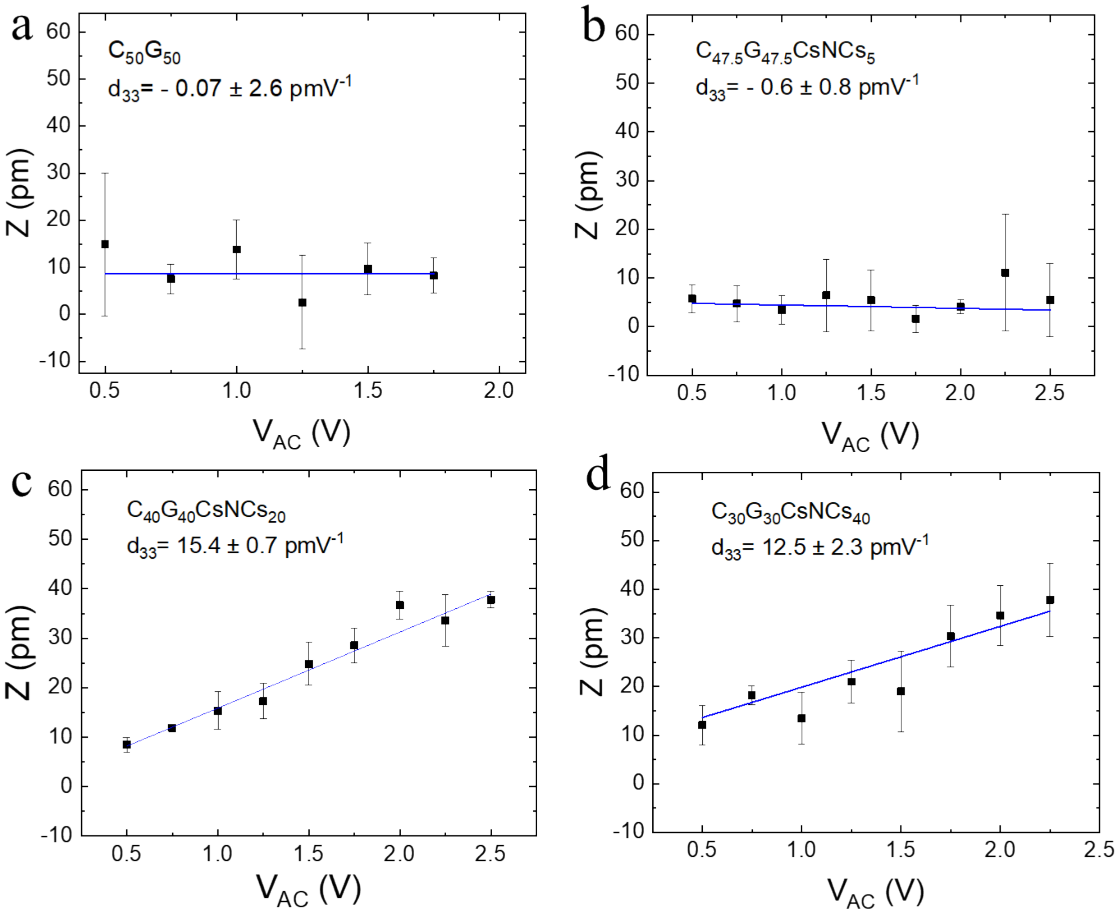

Figure S6. Average on 4 points and piezoresponse Z as a function of V_{AC} fitted linearly for $C_{50}G_{50}$ (a), $C_{47.5}G_{47.5}CsNCs_5$ (b), $C_{40}G_{40}CsNCs_{30}$ (c) and $C_{30}G_{30}CsNCs_{40}$ (d). The slope of the curves, hence d_{33}, is printed on the graphs.

Stress-strain curves for different composition of films

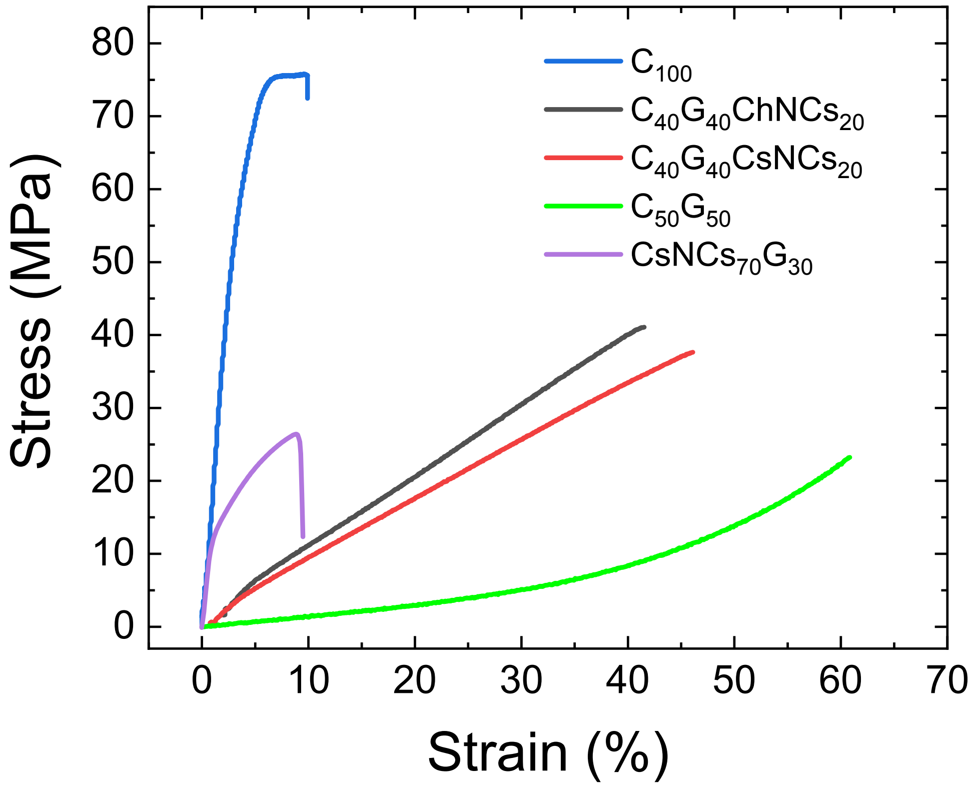

Figure S7. Stress/strain curves of C_{100} (blue curve), $C_{50}G_{50}$ (green curve), $C_{40}G_{40}ChNCs_{20}$ (black curve), $C_{40}G_{40}CsNCs_{20}$ (red curve) and $CsNCs_{70}G_{30}$ (purple curve). Nanocrystals enhance materials rigidity, increasing tensile strength and Young's modulus, highlighting their reinforcing effect as reported in the literature[4]. The addition of glycerol improves film deformability by acting as a plasticizer, as evidenced by the decrease in tensile strength due to reduced intermolecular interactions. These findings are consistent with the literature[1].

X-Ray Diffraction analysis

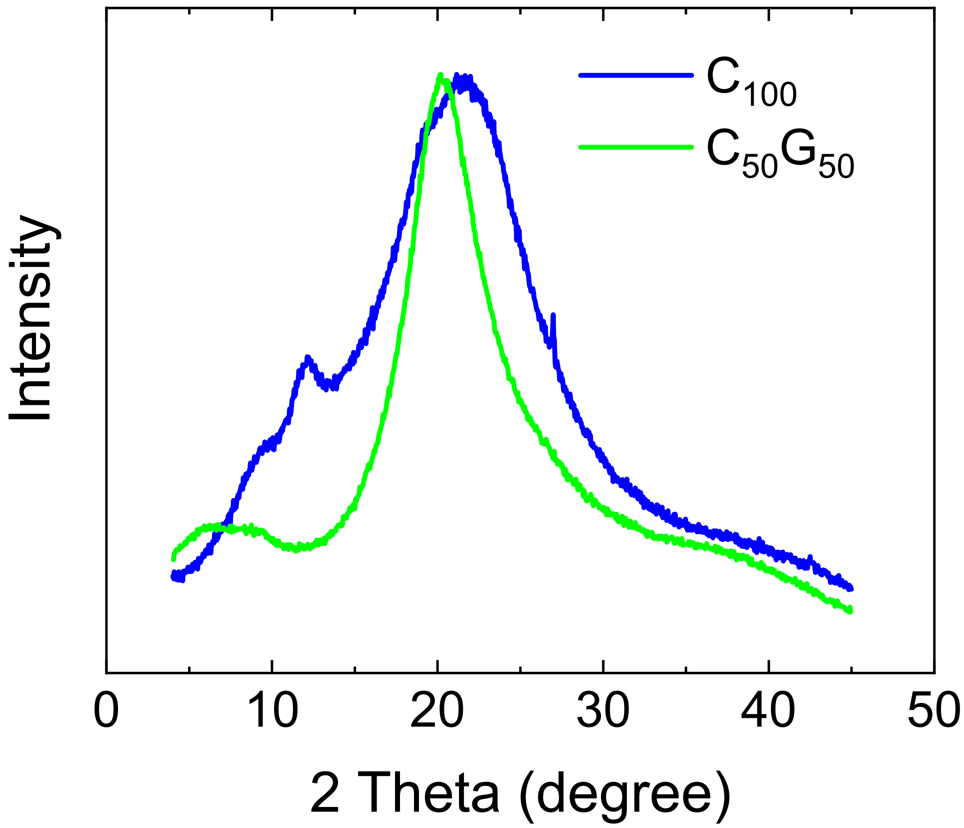

Figure S8. XRD pattern of C_{100} (blue curve) and $C_{50}G_{50}$ (green curve)

Table 1. Manufacturing technology, measuring piezoelectric technique and piezoelectric coefficient d_{33} values for different soft polymers

Material	d_{33} [pmV^{-1}]	Measurement technique	Processing	Reference
Silicone rubber + Barium titanate	22.5	Acquisition Voltage circuit	mixing, moulding polarization and curing.	[5]
Polyacrylonite	40	-	-	[5]
Thermosetting silicone + PZT	60	-	-	[5]
PDMS + PVDF	8	-	-	[5]
PDMS + poly(siloxane-imide)	0.4 to 6.1 (depending on PI %)	PFM	Polycondensation and chemical imidization	[6]
PDMS + PI	0.5 pm /V (pure PDMS); from 17 pm/V to 26 pm/V (depending on PI%)	PFM	Polycondensation	[7]

PVDF + polyacrylonitrile	63	Dynamic mechanical tester	Radical polymerization and freeze-drying processes	[8]
Chloroprene rubber + 60 vol% PZT	244	-	-	[9]
Poly(acrylonitrile butadiene) rubber + 80 vol% PMN	33	-	-	[9]
PDMS + 43 vol % PZT	6.8	-	-	[9]
PDMS + 8 vol % BCZT	31	-	-	[9]
PDMS + 50 vol % PZT	101	-	-	[9]
PDMS + 20-60 vol % PZT	80 - 240	-	-	[9]
CNA-doped polyurethane foams	244	-	-	[9]
PDMS + 33–40 wt% poly [(MMA)-co-(DR1-MA)]	27	-	-	[9]
PDMS + 30 wt% polar polynorbornene	37	-	-	[9]
Polar smectic bent-core liquid crystal material confined in a biocompatible triblock copolymer (BCLC/SIBSTAR composite).	1nm/V (1000 pm/V)	Mirau interferometry	0.5 and 1 mm thick elastomer films were made by solvent casting followed by compression molding under 50 atm pressure that was released at 60 μC, when the liquid crystal component was in the X-phase.	[10]

Table 2. Manufacturing technology, measuring piezoelectric technique and piezoelectric coefficient d_{33} values for different bio-polymers.

Material	Piezoelectric coefficient [pmV^{-1}]	Measurement technique	Processing	Reference
Diphenylalanine (Peptide)	d_{15}= 60	PFM	Self-assembly nanotube	[11]
Collagen fibrils (Protein)	d_{15}= 1	-	-	[12]
Collagen (Protein)	d_{14}= 12	PFM	Use of fibrillary rat tail collagen	[13]
Cellulose NCs (Polysaccharides)	d_{33}= 19.3	-	-	[14]
Lysozyme (Protein)	d_{33}= 6.5	Piezometer	Drop casting	[13]

Cysteine (Amino acid)	d_{22}= 11	-	-	[13]
Glycine (Amino acid)	d_{33}= 4.7, d_{16}= 178	Piezometer	Drop casting	[15]
Silk thin film	d_{33}= 56.7	PFM	Spin coating	[16,17]
Proline Amino acid	d_{25}= 27.5	Impedance/gain phase analyser	Growing of crystals	[13,18]

Table 3. Manufacturing technology, measuring d_{33} technique and piezoelectric coefficient d_{33} values for different chitosan-based materials.

**Material**	**d_{33} [pmV^{-1}]**	**Measurement technique**	**Processing**	**Reference**
Chitosan thin film (thickness = 15 μm)	15.56	PFM	Solvent casting and neutralization	[19]
Chitosan film (thickness = 70 μm)	2.54	PFM	Drop casting	[20]
Chitosan pellets (thickness = 1.3 mm)	18.4	PFM	Manual hydraulic pressure	[21]
Chitosan-poly(3-hydroxybutyrate) (CS-PHB) blend thin films (PHB at 13 wt%) (thickness = 40 μm)	5	PFM	Solvent casting	[22]
Chitosan thin film (thickness = 20 μm)	6	Dynamic force sensor	Solvent casting	[23]
Chitosan-glycine (1:0.8) (thickness= 38 μm)	-	Dynamic pressure using a TIRA shake	Drop casting	[24]
Chitosan - PEDOT nanofibers	-	Pressure and electrochemical system	Electrospinning and H_2SO_4 - treatment for recrystallization	[25]
Chitosan film	-	In-house built setup (shaker for dynamic excitation force)	Solvent casting and neutralization	[26]
Chitosan - Glycerol-Chitin/Chitosan Nanocrystals films	15.4 – 18.7	PFM	Solvent casting	Our work

Table 4. Roughness measurements obtained from PFM scans

**Material**	**Roughness average [nm]**	**Roughness root mean square [nm]**
Chitosan	0.51	0.67
Chit + Gly + CtNCs	5.22	6.49
Chit + Gly + CsNCs	3.51	4.44

ABBREVIATIONS

- **PFM**, Piezoresponse force microscopy;
- **ChNCs,** Chitin nanocrystals;
- **CsNCs**, surface-deacetylated Chitin nanocrystals;
- **C_{100}**, pure Chitosan film;
- **C_{50} G_{50}**, Chitosan (50 wt%) / Glycerol (50 wt%) film;
- **C_{40} G_{40}ChNCs$_{20}$**, Chitosan (40 wt%) / Glycerol (40 wt%) / ChNCs (20 wt%) film;
- **C_{40} G_{40}CsNCs$_{20}$**, Chitosan (40 wt%) / Glycerol (40 wt%) / CsNCs (20 wt%) film;
- **$C_{47.5}$ $G_{47.5}$ChNCs$_{5}$**, Chitosan (47.5 wt%) / Glycerol (47.5 wt%) / ChNCs (5 wt%) film;
- **$C_{47.5}$ $G_{47.5}$CsNCs$_{5}$**, Chitosan (47.5 wt%) / Glycerol (47.5 wt%) / CsNCs (5 wt%) film;
- **C_{30} G_{30}ChNCs$_{40}$**, Chitosan (30 wt%) / Glycerol (30 wt%) / ChNCs (40 wt%) film;
- **C_{30} G_{30}CsNCs$_{40}$**, Chitosan (30 wt%) / Glycerol (30 wt%) / CsNCs (40 wt%) film.

PFM domain switching experiment

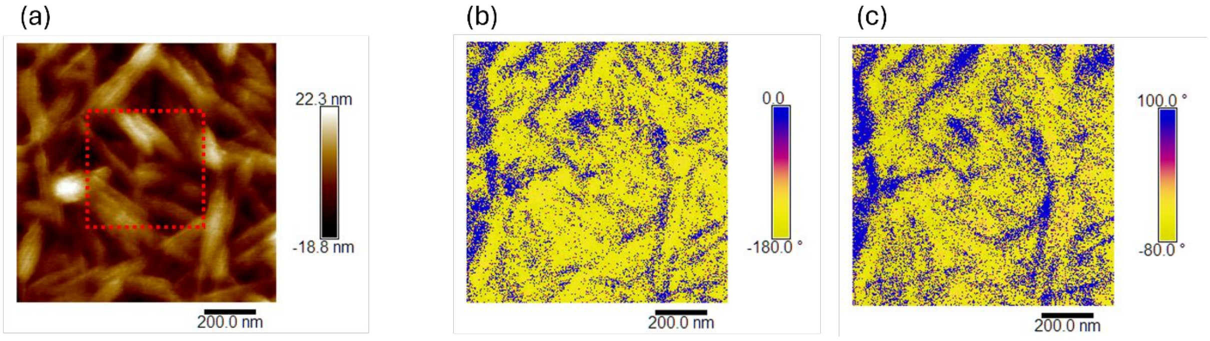

Figure S9 - Box-in-box switched pattern experiment. PFM images of the topography (a) and the out-of-plane phase (a) collected on the surface of C_{40} G_{40} $CsNCs_{20}$ (1 μm × 1 μm area). The red dotted square in (a) shows the 500 nm × 500nm area that was subsequently scanned applying a bias voltage V_{DC}=12 V. (c) PFM image of the out-of-plane phase collected from the extended original area of 1 μm × 1 μm after the application of the V_{DC} bias on the central area. The similar patterns of phase observed in (b) and (c) indicate the absence of polarization domain swithing induced by V_{DC}=12 V, which is the maximum voltage supplied by the AFM equipment.

References

(1) Sole, R.; Buranello, C.; Di Michele, A.; Beghetto, V. Boosting Physical-Mechanical Properties of Adipic Acid/Chitosan Films by DMTMM Cross-Linking. *Int. J. Biol. Macromol.* **2022**, *209*, 2009–2019. https://doi.org/10.1016/j.ijbiomac.2022.04.181.

(2) Kumirska, J.; Czerwicka, M.; Kaczyński, Z.; Bychowska, A.; Brzozowski, K.; Thöming, J.; Stepnowski, P. Application of Spectroscopic Methods for Structural Analysis of Chitin and Chitosan. *Mar. Drugs* **2010**, *8* (5), 1567–1636. https://doi.org/10.3390/md8051567.

(3) Abdolrahimi, M.; Seifi, M.; Ramezanzadeh, M. H. Study the Effect of Acetic Acid on Structural, Optical and Mechanical Properties of PVA/Chitosan/MWCNT Films. *Chin. J. Phys.* **2018**, *56* (1), 221–230. https://doi.org/10.1016/j.cjph.2017.12.018.

(4) Fernández-Marín, R.; Morales, A.; Erdocia, X.; Iturrondobeitia, M.; Labidi, J.; Lizundia, E. Chitosan–Chitin Nanocrystal Films from Lobster and Spider Crab: Properties and Environmental Sustainability. *ACS Sustain. Chem. Eng.* **2024**, *12* (28), 10363–10375. https://doi.org/10.1021/acssuschemeng.4c01205.

(5) Nicolini, L.; Sorrentino, A.; Castagnetti, D. A Soft Piezoelectric Elastomer with Enhanced Piezoelastic Response. *Smart Mater. Struct.* **2023**, *32* (10), 105003. https://doi.org/10.1088/1361-665X/acef81.

(6) Stiubianu, G.-T.; Bele, A.; Bargan, A.; Potolinca, V. O.; Asandulesa, M.; Tugui, C.; Tiron, V.; Hamciuc, C.; Dascalu, M.; Cazacu, M. All-Polymer Piezo-Composites for Scalable Energy Harvesting and Sensing Devices. *Molecules* **2022**, *27* (23), 8524. https://doi.org/10.3390/molecules27238524.

(7) Tugui, C.; Bele, A.; Tiron, V.; Hamciuc, E.; Varganici, C. D.; Cazacu, M. Dielectric Elastomers with Dual Piezo-Electrostatic Response Optimized through Chemical Design for Electromechanical Transducers. *J. Mater. Chem. C* **2017**, *5* (4), 824–834. https://doi.org/10.1039/C6TC05193F.

(8) Guan, Y.; Tu, L.; Ren, K.; Kang, X.; Tian, Y.; Deng, W.; Yu, P.; Ning, C.; Fu, R.; Tan, G.; Zhou, L. Soft, Super-Elastic, All-Polymer Piezoelectric Elastomer for Artificial Electronic Skin. *ACS Appl. Mater. Interfaces* **2023**, *15* (1), 1736–1747. https://doi.org/10.1021/acsami.2c19654.

(9) Owusu, F.; Venkatesan, T. R.; Nüesch, F. A.; Negri, R. M.; Opris, D. M. How to Make Elastomers Piezoelectric? *Adv. Mater. Technol.* **2023**, *8* (15), 2300099. https://doi.org/10.1002/admt.202300099.

(10) Charif, A. C.; Diorio, N.; Fodor-Csorba, K.; Puskás, J. E.; Jákli, A. A Piezoelectric Thermoplastic Elastomer Containing a Bent-Core Liquid Crystal. *RSC Adv.* **2013**, *3* (38), 17446. https://doi.org/10.1039/c3ra41766b.

(11) Kholkin, A.; Amdursky, N.; Bdikin, I.; Gazit, E.; Rosenman, G. Strong Piezoelectricity in Bioinspired Peptide Nanotubes. *ACS Nano* **2010**, *4* (2), 610–614. https://doi.org/10.1021/nn901327v.

(12) Sun, Y.; Zeng, K.; Li, T. Piezo-/Ferroelectric Phenomena in Biomaterials: A Brief Review of Recent Progress and Perspectives. *Sci. China Phys. Mech. Astron.* **2020**, *63* (7), 278701. https://doi.org/10.1007/s11433-019-1500-y.

(13) Ali, M.; Bathaei, M. J.; Istif, E.; Karimi, S. N. H.; Beker, L. Biodegradable Piezoelectric Polymers: Recent Advancements in Materials and Applications. *Adv. Healthc. Mater.* **2023**, *12* (23), 2300318. https://doi.org/10.1002/adhm.202300318.

(14) Sun, B.; Chao, D.; Wang, C. Piezoelectric Nanogenerator Based on Electrospun Cellulose Acetate/Nanocellulose Crystal Composite Membranes for Energy Harvesting Application. *Chem. Res. Chin. Univ.* **2022**, *38* (4), 1005–1011. https://doi.org/10.1007/s40242-021-1252-x.

(15) Guerin, S.; Stapleton, A.; Chovan, D.; Mouras, R.; Gleeson, M.; McKeown, C.; Noor, M. R.; Silien, C.; Rhen, F. M. F.; Kholkin, A. L.; Liu, N.; Soulimane, T.; Tofail, S. A. M.; Thompson, D. Control of Piezoelectricity in Amino Acids by Supramolecular Packing. *Nat. Mater.* **2018**, *17* (2), 180–186. https://doi.org/10.1038/nmat5045.

(16) Joseph, J.; Singh, S. G.; Vanjari, S. R. K. Leveraging Innate Piezoelectricity of Ultra-Smooth Silk Thin Films for Flexible and Wearable Sensor Applications. *IEEE Sens. J.* **2017**, *17* (24), 8306–8313. https://doi.org/10.1109/JSEN.2017.2766163.

(17) Joseph, J.; Singh, S. G.; Vanjari, S. R. K. Piezoelectric Micromachined Ultrasonic Transducer Using Silk Piezoelectric Thin Film. *IEEE Electron Device Lett.* **2018**, *39* (5), 749–752. https://doi.org/10.1109/LED.2018.2816646.

(18) Guerin, S.; Syed, T. A. M.; Thompson, D. Deconstructing Collagen Piezoelectricity Using Alanine-Hydroxyproline-Glycine Building Blocks. *Nanoscale* **2018**, *10* (20), 9653–9663. https://doi.org/10.1039/C8NR01634H.

(19) De Marzo, G.; Mastronardi, V. M.; Algieri, L.; Vergari, F.; Pisano, F.; Fachechi, L.; Marras, S.; Natta, L.; Spagnolo, B.; Brunetti, V.; Rizzi, F.; Pisanello, F.; De Vittorio, M. Sustainable, Flexible, and Biocompatible Enhanced Piezoelectric Chitosan Thin Film for Compliant Piezosensors for Human Health. *Adv. Electron. Mater.* **2022**, 2200069. https://doi.org/10.1002/aelm.202200069.

(20) De Marzo, G.; Desmaële, D.; Algieri, L.; Natta, L.; Guido, F.; Mastronardi, V.; Mariello, M.; Todaro, M. T.; Rizzi, F.; De Vittorio, M. Chitosan-Based Piezoelectric Flexible and Wearable Patch for Sensing Physiological Strain. In *The 8th International Symposium on Sensor Science*; MDPI, 2021; p 12. https://doi.org/10.3390/I3S2021Dresden-10124.

(21) Praveen, E.; Murugan, S.; Jayakumar, K. Investigations on the Existence of Piezoelectric Property of a Bio-Polymer – Chitosan and Its Application in Vibration Sensors. *RSC Adv.* **2017**, *7* (56), 35490–35495. https://doi.org/10.1039/C7RA04752E.

(22) Toalá, C. U.; Prokhorov, E.; Barcenas, G. L.; Landaverde, M. A. H.; Limón, J. M. Y.; Gervacio-Arciniega, J. J.; De Fuentes, O. A.; Tapia, A. M. G. Electrostrictive and Piezoelectrical Properties of Chitosan-Poly(3-Hydroxybutyrate) Blend Films. *Int. J. Biol. Macromol.* **2023**, *250*, 126251. https://doi.org/10.1016/j.ijbiomac.2023.126251.

(23) Hänninen, A.; Sarlin, E.; Lyyra, I.; Salpavaara, T.; Kellomäki, M.; Tuukkanen, S. Nanocellulose and Chitosan Based Films as Low Cost, Green Piezoelectric Materials. *Carbohydr. Polym.* **2018**, *202*, 418–424. https://doi.org/10.1016/j.carbpol.2018.09.001.

(24) Hosseini, E. S.; Manjakkal, L.; Shakthivel, D.; Dahiya, R. Glycine–Chitosan-Based Flexible Biodegradable Piezoelectric Pressure Sensor. *ACS Appl. Mater. Interfaces* **2020**, *12* (8), 9008–9016. https://doi.org/10.1021/acsami.9b21052.

(25) Du, L.; Li, T.; Jin, F.; Wang, Y.; Li, R.; Zheng, J.; Wang, T.; Feng, Z.-Q. Design of High Conductive and Piezoelectric Poly (3,4-Ethylenedioxythiophene)/Chitosan Nanofibers for Enhancing Cellular Electrical Stimulation. *J. Colloid Interface Sci.* **2020**, *559*, 65–75. https://doi.org/10.1016/j.jcis.2019.10.003.

(26) Hänninen, A.; Rajala, S.; Salpavaara, T.; Kellomäki, M.; Tuukkanen, S. Piezoelectric Sensitivity of a Layered Film of Chitosan and Cellulose Nanocrystals. *Procedia Eng.* **2016**, *168*, 1176–1179. https://doi.org/10.1016/j.proeng.2016.11.397.
